# The structure of performance and training in esports

**DOI:** 10.1371/journal.pone.0237584

**Published:** 2020-08-25

**Authors:** Eugen Nagorsky, Josef Wiemeyer

**Affiliations:** Institute of Sport Science, Technical University Darmstadt, Darmstadt, Germany; São Paulo State University (UNESP), BRAZIL

## Abstract

Esports as the competitive play of digital games has gained considerable popularity. However, a comprehensive framework for esport training is still missing. In this paper, a performance model integrating insights from game research and sport science is developed. Based on this model, an online questionnaire was designed and applied to investigate training in different esports regarding relevant competencies and training areas. Overall, 1,835 esports players voluntarily participated in the study. Age ranged from 13 to 47 years (*M* = 20,9; *SD* = 4,5), and males clearly dominated (95%). Furthermore, the mean weakly playing time was 20.03 hours (SD = 15.8). Training occupied 38.85% (7.75 h) of the playing time on average. On the one hand, the results reveal game-specific competence and training structures in the five esports selected for the study (Starcraft II, League of Legends, Rocket League, FIFA, and Counter Strike). On the other hand, the factor structure of competencies closely resembles the esports performance model. As a conclusion, esports training methods should always consider the specific competence profile of the respective esports game.

## Introduction

The competitive play of various digital games (esports) has gained considerable popularity. While in 2015 the esports audience was estimated at 235 million, in 2018 it already reached 395 million. In the next few years, a continued growth in popularity is expected [1,2]. Esports is receiving increasingly more recognition in sports. In China, esport has been already considered a sport since 2003. Esport was an item in Asian indoor martial arts games in 2017 and will be an official competitive sports item in the 19th Asian games in Hangzhou 2022 [3]. In the USA, esports players have been considered professional athletes since 2013 [4]. However, there still is no general agreement, how esports relates to sports. There are certain elements within esports, that are similar to traditional sports [5]. The infrastructure of esports can be compared to traditional team sports like soccer, ice hockey, or basketball. Professional esports players are not only getting paid by sponsors but are signed by professional esports organizations and clubs. The existence of player contracts, trade periods and buyouts indicates phenomena that are very well known in traditional sports. Therefore, it is not surprising that professional sports clubs like Paris Saint Germain, Manchester City, Ajax Amsterdam, Golden State Warriors, and Philadelphia 76ers are increasingly getting involved in esports to extend their recognition (data source: https://liquipedia.net/).

Esports can be differentiated into specific genres such as Multiplayer Online Battlefield Arena (MOBA), First Person Shooters (FPS), Real Time Strategy (RTS) and sport simulations [5]. In every genre, there are different digital games, with different mechanics and competition rules that have to be mastered by the players. To achieve and maintain the maximum level of performance, the players have to continuously exercise and improve or maintain their skills and abilities. Particularly in the area of training and performance improvement, a distinctive difference between professional sports and professional esports exists. While training of professional sports athletes is based on well-established scientific research, esports training is not yet in the full scope of sports science. With some exceptions [6], there is little research addressing skill and performance improvement in esports. Regarding the training of esports athletes, there are several key questions: Which skills and abilities are required to succeed in esports? Which esports skills compare to skills required in traditional sports? Are different skills needed for different esports disciplines/games? How do esports athletes currently exercise?

Considering the lack of theory and empirical studies, this paper aims to create a basic framework for future training research in esports and to provide a perspective on esports performance that integrates sports science and (digital) game science. It adds insights into the structure of esports performance as well as current training methods of esports athletes and identifies the most important components in representative esports disciplines and genres. The paper is structured as follows: First, we analyse the performance models in game and sports science and propose an integrative framework of esports performance. Second, we describe an empirical study addressing the structure of performance and training in esports. Finally, we discuss the results and draw conclusions for further research.

## Performance structure of esports–theory

In this section, performance models in game and sport science are introduced and analysed regarding common components. In a second step, a unified framework for esports is proposed integrating the insights from the game and sport domain.

### Models of game competence

In today´s game science, models of game competence are often used to describe certain competencies or skills relevant to digital games. These competence models can also be used as a basis to identify appropriate methods to improve player performance in video games and esports. In game science, there are several models, concepts and theories as well as single studies addressing competencies of video game players.

**Fig 1:**
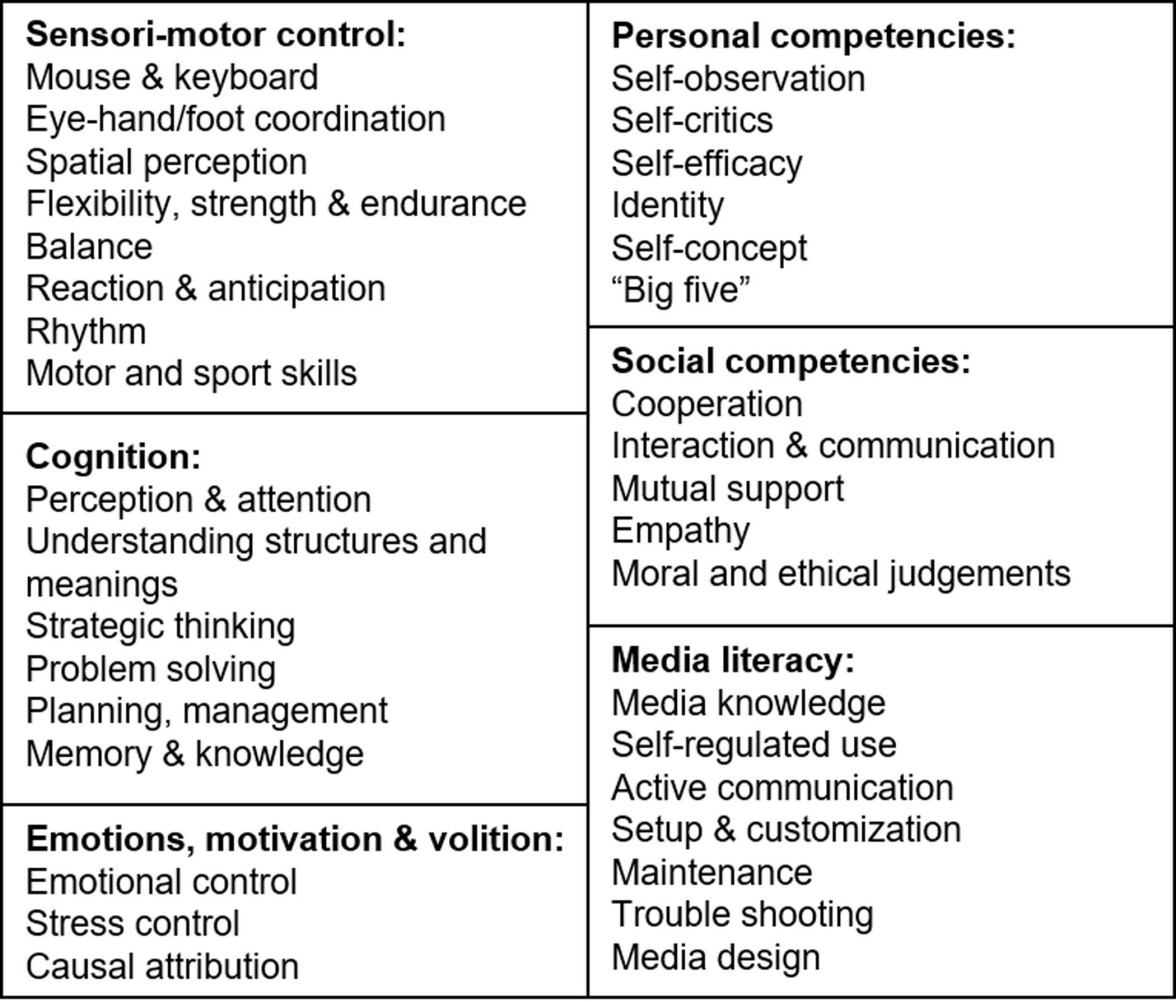
Competence model of digital games (according to [9] and [11]).

Comprehensive models of game competence are provided by Kraam-Aulenbach [7,8] and Wiemeyer and Hardy [9]. The model proposed by Kraam-Aulenbach [7,8] describes gaming as a problem-solving process. She identifies a problem-solving mind, inductive skills, spatial imagination, eye-hand coordination and social competencies as the central competencies of digital games. The competence model proposed by Wiemeyer and Hardy [9] is an extension of the model published by Gebel et al. [10]. As can be seen in Fig 1, six dimensions of competencies are distinguished: Sensori-motor control, cognition, personal competencies, emotion and volition, social competencies, and media literacy. Several competencies can be assigned to these dimensions. While the sensorimotor, cognitive, emotional/volitional and personal competencies mostly describe physical and psychic abilities that are required to play video games, social competencies focus on human interaction and communication. Media literacy describes the ability to handle electronic devices required to install, customize, maintain and play games. All competencies mentioned in the models are potentially relevant in the competitive environment of esports professionals as well.

Empirical studies exist that confirm the effect of digital games on competencies claimed by the above-mentioned models. For example, Li et al. [12] reveal transfer effects of 5 to 10 hours of engagement in action video games on the hand-eye coordination of the player. Green and Bavelier [13] find improvements on spatial perception after playing an ego-shooter game for 30 days. Lager and Bremberg [14] notice positive effects on spatial perception and reaction time in several studies. The experimental groups played games for a total of 14 to 33 hours over a period of a few months.

### Models of sport performance

**Fig 2:**
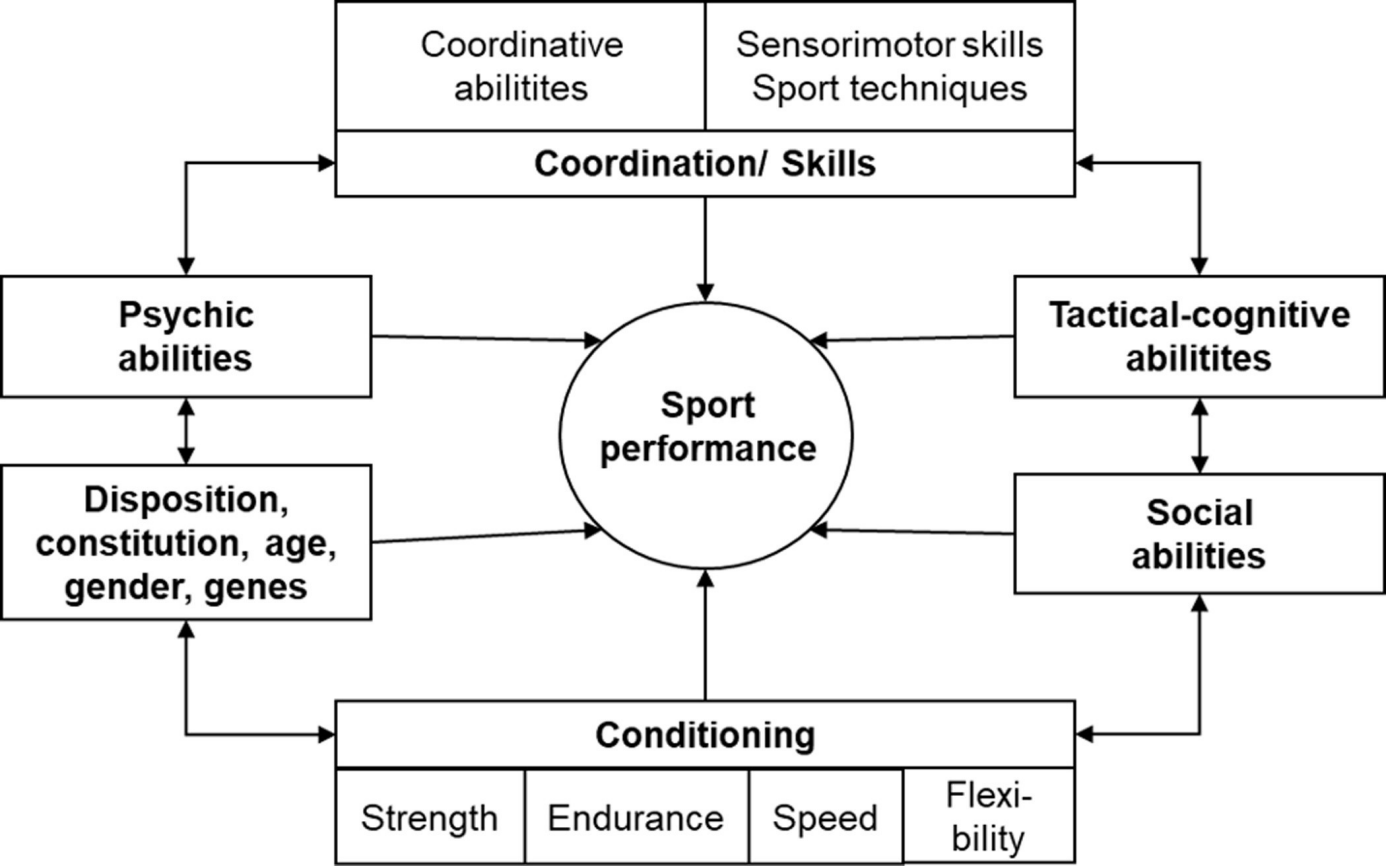
Generic model of sport performance [15].

In sports, various performance models exist. In particular, generic models of human performance play an important role in training science, since this discipline of sport science aims at systematically and sustainably influencing sport performance by training interventions. A prototypical model is illustrated in Fig 2 In this model, six main “building blocks” of performance are distinguished:

- Coordination/skills
- Condition
- Cognitive-tactical skills
- Psychic abilities
- Social abilities
- Disposition, constitution, age, gender, and genes

#### Coordination/skills

Sensorimotor coordination primarily regards features of information processing when performing movements. This concept comprises two important sub-components, i.e., specific sensori-motor skills such as running, jumping or throwing techniques which are typical for particular sports, and generic coordinative abilities, i.e., skill-independent competencies such as balance, spatial orientation, and sensory discrimination as well as precision and speed.

Regarding sensori-motor skills, different types are distinguished depending on the situational demands on skill execution. Regarding situations, three types can be distinguished: static situations, and situations changing in an expected or unexpected way. When responding to these different situations, the respective execution must be kept constant or has to be adapted.

Regarding generic coordinative abilities, numerous abilities are proposed that influence the control, adaptation and learning of motor skills: Motor imagery, motor memory, balance, anticipation/reaction, spatial orientation, rhythm, dexterity, agility, open- and closed-loop control, eye-hand and eye-foot coordination [16].

**Fig 3:**
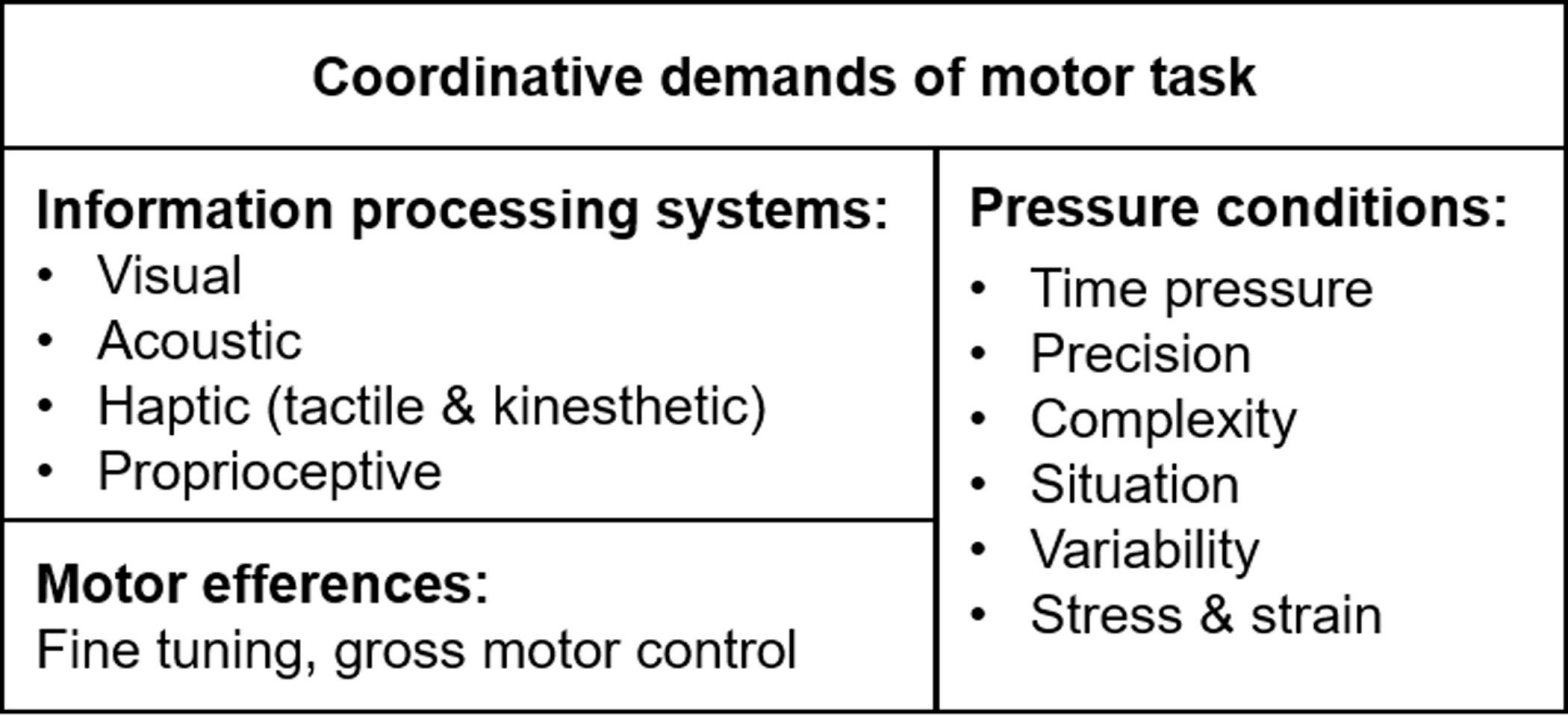
Coordination model of [17].

Another approach to sensori-motor coordination distinguishes between informational demands of the tasks regarding sensory and motor processing as well as various pressure conditions influencing the control of movements ([17]; see Fig 3).

#### Condition

The condition category includes four components regarding energetics of human movements: Endurance, strength, speed, and flexibility [18,19].

Endurance denotes the ability to perform physical activities of certain duration without loss in performance and to recover fast from physical load (strain). Different categories of endurance are distinguished with respect to metabolism (aerobic, anaerobic-lactacide, anaerobic-alactacide), proportion of activated muscle mass (local, regional, global), type of muscle work (static, dynamic) and duration (short, middle and long term).

Strength is defined as the ability to produce as much force as possible in order to overcome a resistance (concentric or positive work), hold a joint position (isometric or static work) or give way to supramaximal load (eccentric or negative work). Beyond type of muscle action maximum force is distinguished from power and force endurance.

Speed denotes the ability of the sensori-motor system to react to certain events and to perform movements as fast as possible. Speed abilities can be distinguished into cyclic and discrete actions as well as reactions. Speed can be specific or elementary.

Flexibility is defined as the ability of the sensori-motor system to perform movements with the required, i.e. optimal, range of motion (ROM). Flexibility is distinguished into active versus passive as well as static versus dynamic ROM.

#### Cognitive-tactical abilities

In many sports, cognitive-tactical skills are required. For example, in sport games it is important to perceive and judge the current situation as early and fast as possible in order to take and implement the adequate decisions. Tactical abilities are distinguished into individual, group and team tactics. Tactical abilities heavily depend on perception, decision making and creativity as well as executive functions such as working memory, attention, and multitasking [20–22].

#### Psychic abilities

When it comes to psychic abilities, beyond cognitive factors mentioned in the previous section, further factors are considered by training science, such as motivation, emotion and volition as well as personality (e.g., the Big Five). For example, achievement motivation, i.e. motivation to meet challenging standards, emotional stability and modes of control (e.g., state versus action orientation; [23]) have a substantial influence on performance.

In addition, personality features may be associated with successful performance. There is substantial scientific evidence that “personality traits relate to long-term athletic success, interpersonal relationships, and athletes’ psychological states before, during, and after competitions” [24], for example the “big five”, i.e., extraversion, agreeableness, openness, neuroticism, and conscientiousness. Especially conscientiousness, tough-mindedness, neuroticism, anxiety, and extraversion contribute to long-term performance [24–26].

#### Social abilities

When playing in teams and against teams, social abilities such as communication, cooperation and collaboration are important factors influencing performance. Many social abilities are influenced by personality [24].

#### Disposition, constitution, age, gender, and genes

Finally, the sixth group of factors indirectly influence performance in sports: disposition, body constitution, age, gender, and genes. Due to the fact, that these factors cannot be directly influenced by training, they will not be addressed in this paper.

### Integrative model

**Fig 4:**
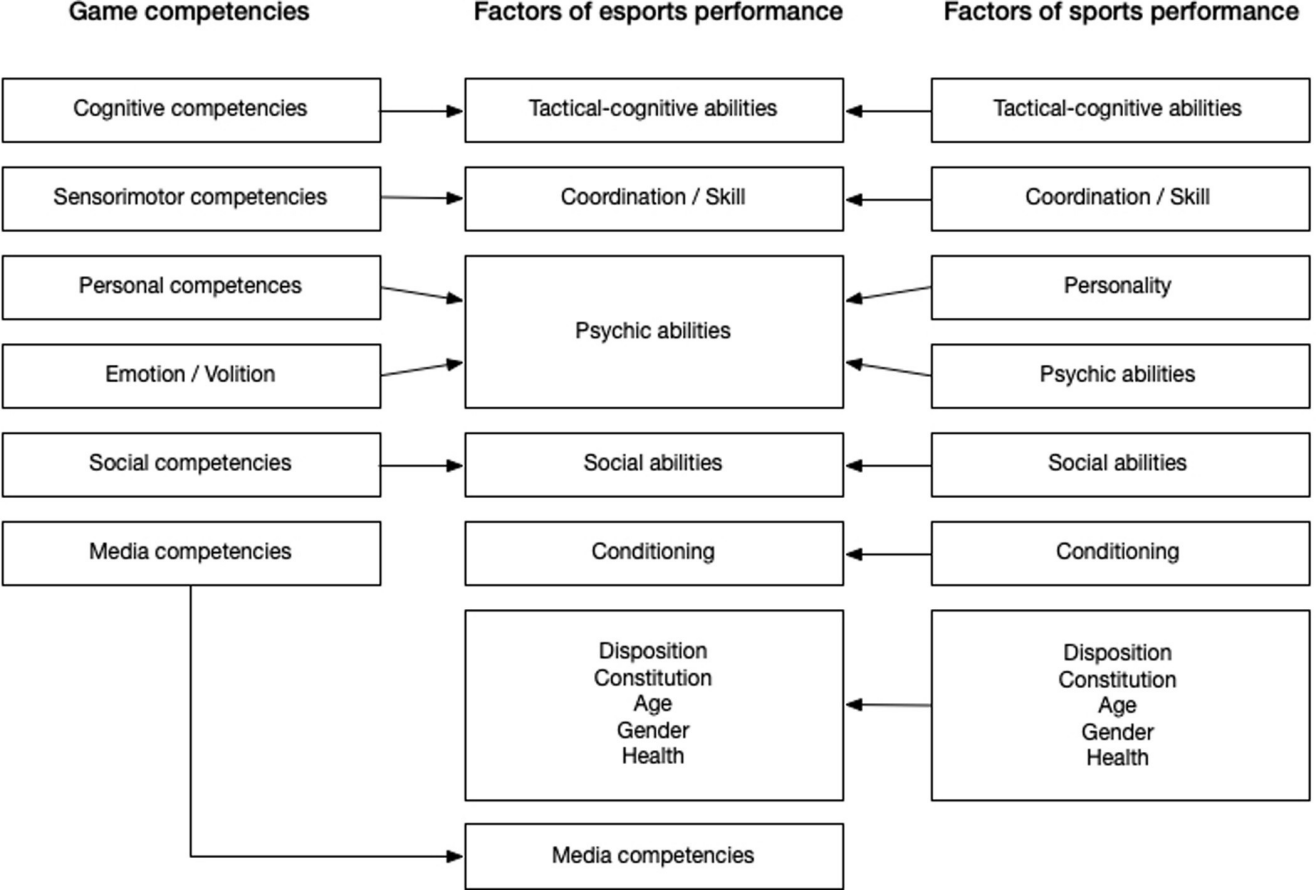
Integrative model of esports performance.

When comparing the competence model of games (section Models of game competence) with the performance model of sports (section Models of sport performance), distinctive similarities are recognizable. Based on the game and sports views, an integrative model of esports performance was derived (Fig 4). In the following, the components of the integrative model will be discussed in relation to esports (see also [5]). Note that, as in real sports, “each game has different goals, rules, and affordances, in different settings, and with different control schemes” [27]. Therefore, the specific performance profile may vary according to the specific structure of the respective esports game and competition.

#### Tactical-cognitive abilities

Just like in sports, tactical and cognitive abilities affect the performance in esports. Strategy games, for example, require the abilities of strategic thinking and decision making based on knowledge induced from experience and rules deduced from theories or models [7]. Gebel et al. [10] proposed a catalogue of cognitive requirements that represents the broad range of the requirements of digital games (Table 1).

**Table 1:** Cognitive requirements catalogue [10].

Domain	Requirements
Memory and concentration	• Memory of game commands, functions and progressions • Concentration • Simultaneous processing of information
Inferential thinking	No specification
Action-planning	• Situation and task analysis • Exploration of action possibilities • Thinking through action steps • Simultaneous planning and initiation of actions
Dealing with complexity	• Complexity of rules • Number of relevant elements and factors • Degree of the connection of relevant elements and factors

To perform at the highest level in esports, the players must meet all requirements as effectively as possible. Therefore, tactical-cognitive abilities are essential components of esports performance. Since esports games are often team-based, not only single-player but also team tactics play an important role in many esports games. As a consequence, training in esports clearly concentrates on this factor [6].

#### Coordination/skill

**Fig 5:**
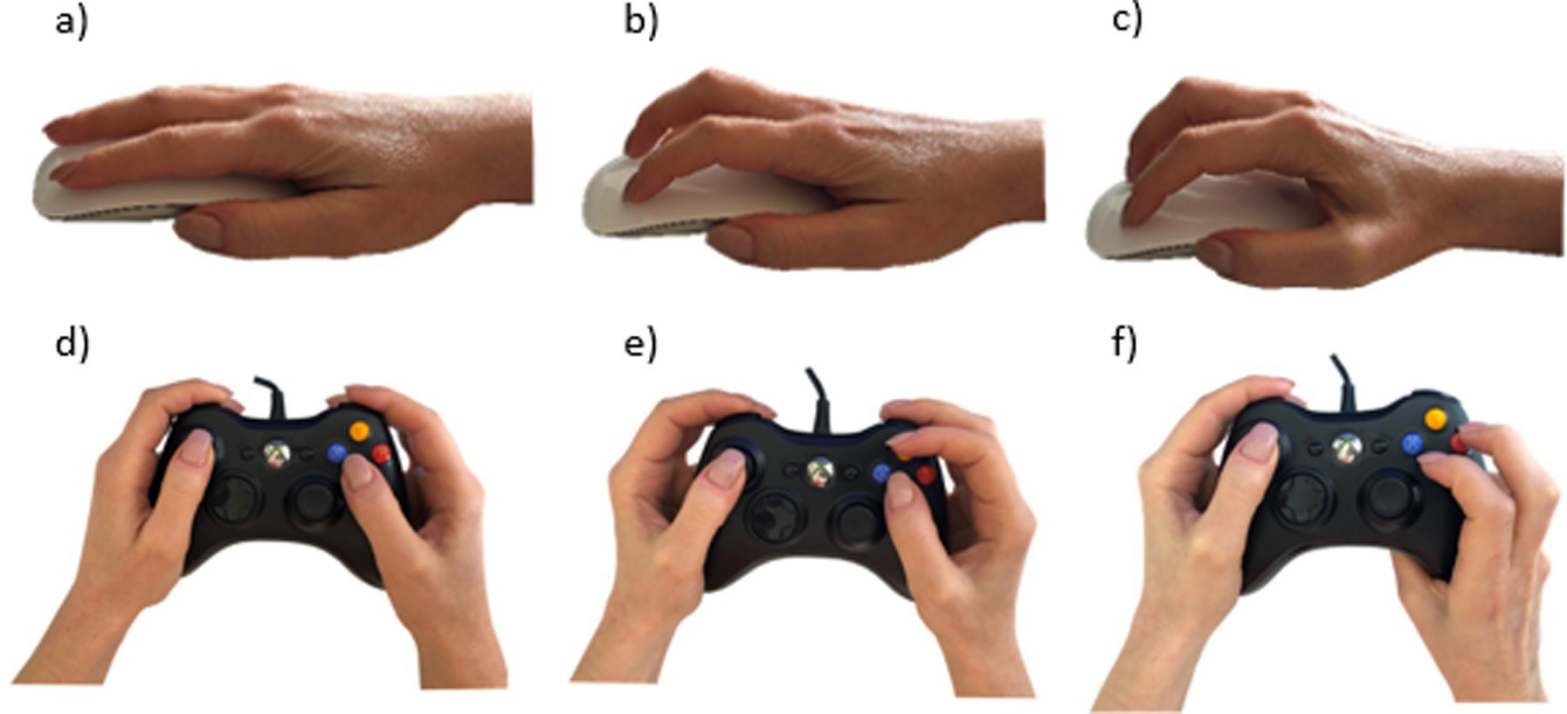
Selected grip techniques in gaming (copyright: Authors). Top row: mouse operations—a—palm; b—claw; c—fingertip; bottom row: gamepad operations d—normal; e—Rocket League; f—action/fight.

Regarding esports, both specific and generic components play an important role. A game is controlled by specific sensori-motor actions (skills) on interfaces and sensors such as hand-mouse, finger-keyboard or hand-joystick interactions as well as body-camera or body-force-platform interactions [28]. Input devices such as mouse, gamepad, and keyboard, have to be operated in a specific way (for examples, see Fig 5) to move avatars, change or use weapons or steer vehicles. Therefore, esports includes skilful physical interactivity [29], however, adapted to the specific perceptual and sensori-motor conditions of the virtual world [28]. Either movements of hands and fingers (”manual dexterity”) or body movements are used for skilful and goal-directed interactions.

One of the most important generic coordinative competencies which is deemed relevant to esports is spatial abilities. Players have to perceive their own position as well as the position of other players, avatars, or manipulanda. The construct of spatial abilities includes several components such as the perception of static or dynamic objects in different spatial reference systems [30]. Several studies provide evidence for a training effect of video gaming on spatial abilities [31] as well as reaction (Reviews of empirical studies: [14,32,33]; video analysis of gameplay: [34,35]; interview studies: [27]). Furthermore, the ability to predict actions and events in a game (i.e., anticipation) is often mentioned as an important component of successful gaming [27]. Furthermore, the ability to combine single movements of fingers, hand, arms, or body parts also plays an important role in esports. In particular, synchronous and sequential coordination have to be performed in order to skilfully interact with the game. Another ability which is important for esports is adaptation. In esports, situations change rapidly and often unexpectedly; this requires the players to flexibly and rapidly adapt own movements to the new situation. When the spatio-temporal coupling of game events and own actions is required, rhythmic abilities play an important role. Finally, proprioceptive discrimination is required for accurate and precise haptic interaction with the input devices.

Considering the informational demands in esports in relation to the model proposed by Neumaier [17], the visual, acoustic, and haptic systems play an important role (Table 2).

**Table 2:** Contribution of selected sensory systems to performance in esports.

System	Contribution (examples)
Visual system	Scene, motions and actions of other players and avatars, change of conditions such as brightness, colour, or shapes
Acoustic system	In-games sounds, communication in the game and with teammates
Haptic system	Position of hands and fingers, force on keyboard, mouse or gamepad, speed of finger movements

Regarding pressure conditions, the following dimensions are distinguished in the Neumaier model: time pressure, precision pressure, situational pressure, complexity pressure and stress-strain pressure. In Table 3, pressure-related demands in esports are illustrated. Particularly, precision of movement is addressed by esports training [6].

**Table 3:** Demands of various pressure conditions in esports.

Pressure condition	Demands (examples)
Time	Execution of perception, decision, and action within limited time; fast reactions to visual, acoustic or haptic stimuli; synchronous and sequential actions
Precision	Accurate and precise movements of mouse, hitting the correct keys and buttons
Situation	Variability and complexity of situations in the game
Complexity	Simultaneous and/or sequential coordination of movements; number of hands, fingers, and movements to be coordinated
Stress and strain	Physical and psychic stress/strain regarding muscles (fingers, hand, arm) as well as cognition, perception, motivation, volition, and emotion

Again, the assumption is plausible, that coordinative and sensori-motor abilities are two of the key factors for performance in esports and therefore should be an integral part of training.

#### Psychic or mental abilities (including personality)

Due to particular competition-induced pressure, esports players have to be emotionally stable and flexible while acting fast, accurately and precisely. Among others, emotional stability and flexible actions depend on the players’ personality traits. An important characteristic for success in esports is overcoming barriers of optimal performance [36]:

- Ineffective attentional control
- Negative consequences of mistakes
- Going on tilt and being harassed
- Limited ability to regulate emotions
- Dwelling on past performances
- Trouble performing under pressure
- Confidence issues
- Inability to repeat flow experience
- Inadequate physical and mental preparation

Just like professional sports athletes, esports players are exposed to stress situations. Dealing with disappointment, performance pressure and fear of failure is therefore part of esports as well.

To succeed in esports, achievement-motivated acting is crucial. Lee and Schoenstedt [37] conclude, that competition and striving to improve individual skills are important motivational factors to play at the highest level. A competitive personality, with the desire to face challenges, is therefore common in esports [38]. For these reasons, mental factors are an important element for success in esports and should always be considered in training.

#### Social abilities

The ability to communicate, collaborate and cooperate within a team is an important component of successful performance in esports [27]. Besides communication, team structure and dynamics as well as teamwork were also found to be important [39,40]. With regard to competition, teammates and viewers, aspects of social behaviour and responsibility are relevant. Reliability, behaviour inside and outside the game after winning or losing, is of high value in professional esports and can have considerable effects on individual and team performance. As a consequence, team cohesion and especially communication is a substantial part of esports training [6].

#### Condition

As mentioned above, the condition category includes four components regarding energetics of human movements: Endurance, strength, speed, and flexibility.

In esports, on the one hand fast movements are required to control the game and on the other hand competitions can take many hours. Therefore, local anaerobic-alactacide endurance in fingers, hands and arms is required as well as global aerobic endurance.

Strength abilities do not seem to play an important role in esports. However, maximal strength is an important determinant of speed. Therefore, this specific subtype of strength may contribute indirectly to game performance. In addition, to control posture, force endurance of the trunk muscles is required. Reactions as well as cyclic and discrete actions play a substantial role in esports. Fast reactions and fast movements constitute the success in many game situations.

In esports, flexibility does not seem to be a factor limiting game performance in general. However, due to the repetitive execution of movements within spatial constraints and prolonged postures, e.g., in a sitting condition [6], specific flexibility exercises may be useful to compensate for these rather unbalanced movement situations. Furthermore, in order to perform skilled movements of the fingers and hands, a certain degree of flexibility may be useful.

Regarding conditioning factors in eSport, global aerobic endurance, local anaerobic-alactacide endurance as well as selected speed abilities seem to be most important. Players have to move quickly according to particular events in the game. On the other hand, strength and flexibility may not be as important. Interestingly, athletes engaged in esports agree that physical fitness has a positive influence on esports performance [6]. As a consequence, more than 60% are physically active for more than 2.5 hours per week, whereas only 28.4% are engaged in regular esports-specific training [6].

#### Constitution

Just like in sports, many constitutional factors contributing to performance, such as dispositions, constitution, age, and gender, cannot be directly influenced by training and will therefore not be addressed in this paper. However, health state can be supported by adequate behaviour, e.g., nutrition, sleep, relaxation, and physical activity.

#### Media competencies

Media competencies are a fundamental prerequisite for practising esports. To play games the players must be able to handle the relevant media (computer, console, mobile phone). Basic technical skills must be present (e.g., installing the game, maintaining the operation system, managing internet connections etc.). The player has to modify the game or the devices used (e.g., adjust game settings, set mouse speed). Knowledge of menu structures, how to deal with communication options (e.g., chat, headset) and orientation in the game environment can also be important in esports [10]. Another important media competence is dealing with technical problems (e.g., acquiring information to solve problems).

## Training structure of esports–empirical evidence

In this section, the integrated model of esports performance proposed in the previous section will be used to analyse the performance requirements and training behaviour of esports players in various esports titles. We will introduce related work, the methods used in our study and will present the results as well as discuss them.

**Fig 6:**
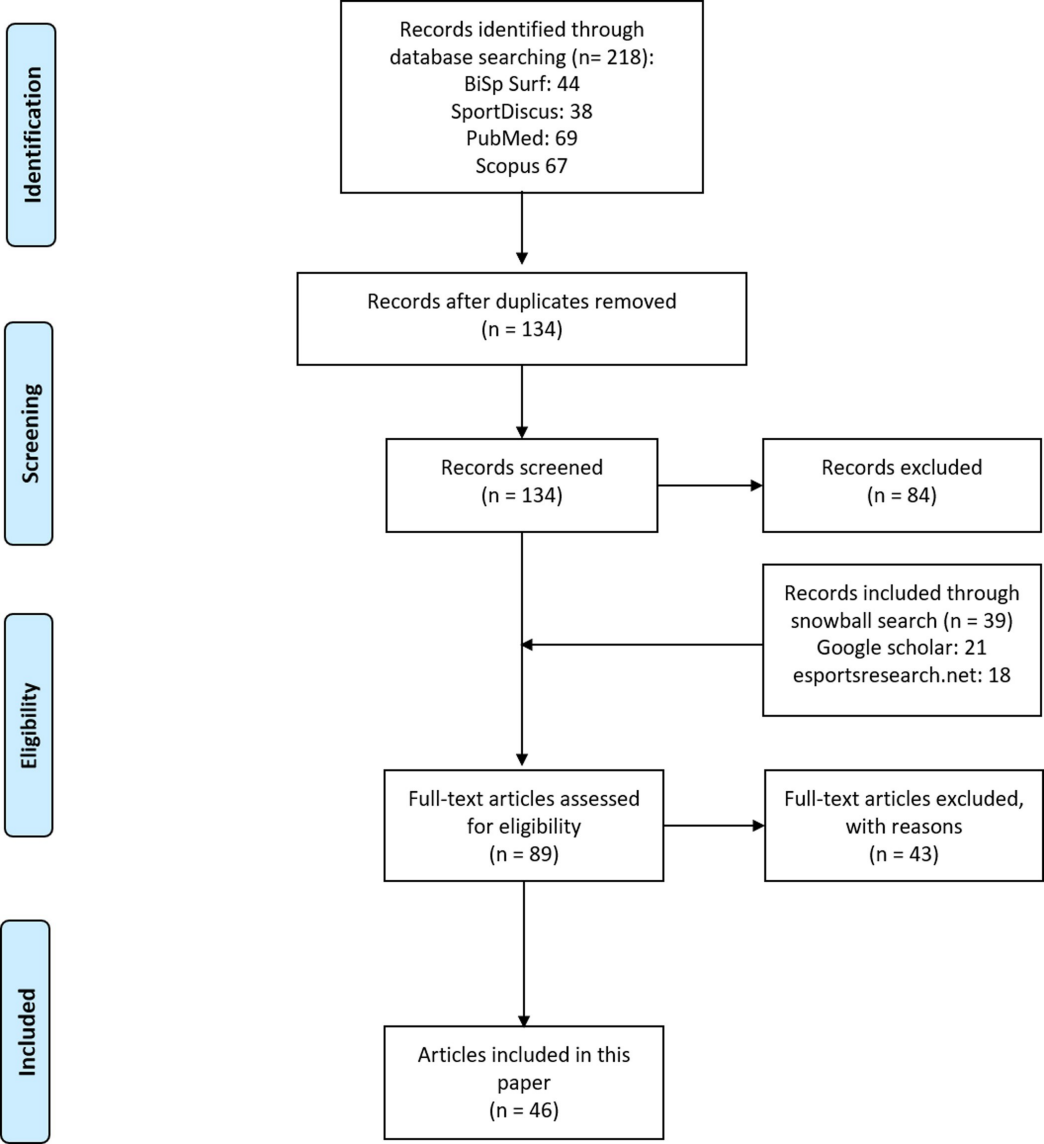
PRISMA-flow chart of systematic literature search.

Relevant studies were located via systematic search shown in Fig 6. The literature search was carried out in four databases (BISp-Surf, SportDiscus, PubMed, Scopus). The publications were selected according to the relevance to training and performance in esports. Articles with relevant insights on the requirements, professional practice and skills/competencies in esports were also considered. In the first search phase, 134 relevant publications were found. After the analysis of the titles and abstracts, 81 publications turned out to be irrelevant; in addition, 3 titles without empirical data were removed. Next, the remaining 50 articles were screened; furthermore, a snowball-search with Google Scholar and a supplementary search on https://esportsresearch.net/literature-on-esports/ (last check on December, 10 2019) was performed. By this strategy, 45 articles were added. After the full-text assessment of 89 articles, 43 articles were excluded because of lack of eligibility. Overall, 46 articles were used in this research.

### Structure of performance

The great majority of existing studies addresses selected components of esports performance (Table 4). With regard to performance, team cohesion, communication, non-verbal communication and team dynamics were examined [39–41]. From a psychological point of view, motivational factors, obstacles encountered by professional players and mental techniques were investigated regarding performance in esports [36–38]. Cognitive and sensori motor aspects of esports such as memory, hand-eye coordination, mental rotation, action speed, game sense, and technique/skill were analysed in some studies [27,34,42–47].

**Table 4:** Studies addressing selected components of esports performance.

Category	Feature	Evidence
**Team performance**	Team cohesion	Algesheimer (2011)
	Communication	Algesheimer (2011)
	Non-verbal communication	Leavitt et al. (2016)
	Team dynamics	Wanyi (2018)
**Psychic**	Mental techniques	Himmelstein et al. (2017)
	Mental barriers	Himmelstein et al. (2017)
	Motivational factors	Lee and Schoenstedt (2011), Weiss & Schiele (2013)
**Cognition / Sensori Motor**	Memory	Bonny & Castaneda (2016), Bonny et al. (2016)
	Hand-Eye coordination	Bowman et al. (2013), Khromov et al. (2018)
	Mental rotation	Bowman et al. (2013)
	Action Speed	Khromov et al. (2018), Lewis et al. (2011)
	Game sense	Fanfarelli (2018)
	Technique/Skill	Fanfarelli (2018), Reeves et al. (2006), (2007), Slacek Brlek (2014)

### Structure of training

Regarding the training in esports, some studies exist that provide insight into the structure of training. In a survey of 1,319 esports players, Adamus [48] examined learning processes in esports. They found, that distinct attributes, i.e., the ability to work in a team, concentration and anticipative thinking, are considered to be most important in esports. The players’ mean engagement in training was 14 hours per week. Kari and Karhulahti [49] interviewed 115 elite esports players and found an average training time of 5.28 hours per day (approximately 37 hours a week). In addition, the authors found that most of the players (55.6%) believed in a significant positive relation between physical training and performance in esports. The difference to the study of Adamus regarding training engagement can be explained by the sampling. While Adamus focused on a broad audience, Kari and Karhulahti focussed on elite esports players. In a survey of 1,200 German esports players, Froboese et al. [6] found that players play 25 hours a week on average (hobby level: 21.8 h/wk; professional level: 27.7 h/wk; amateur level: 28.5 h/wk). Game-mechanics, tactics, communication and movement precision were identified as the main contents of training.

The existing studies show a great heterogeneity. This may be on the one hand due to method-related issues (e.g., questionnaires and sampling) and on the other hand to the heterogeneity of the esports domain. To investigate training, the differentiation between playing time and training time is highly relevant. The training time stands for the deliberate effort of systematically improving esports-related skills and abilities, while the playing time may focus primarily on fun, team experience or outcomes.

Furthermore, esports game titles show a great variety. As stated in the beginning, esports can comprise different genres and different game modes. Every genre as well as different esports within the same genre have their specific goals, rules and requirements in different settings and different control schemes [27]. Game modes consist of single and team modes. The teams in esports usually consist of 3 to 5 members. Furthermore, esports are played on different platforms (mostly on console, notebook or desktop computer, but also on mobile phones) and with different control devices (e.g., mouse, keyboard, gamepad). Since there are different requirements in every single genre and esport, the conclusion is justified, that different abilities and training methods are required to increase the skill level and performance of a player. To analyse and possibly improve the training in esports, it is important to analyse each esport separately.

## Method

### Selection of esports games for the study

In Table 5, the games selected for the study presented in this paper are characterized by genres, platform, play modes, and controls. In the sports genre, two titles were chosen deliberately to determine possible differences between single and team games within the same genre.

**Table 5:** Esports games selected for the study.

Genre	Game	Platform	Controls	Play mode
RTS	Starcraft II	PC	KBM	Single
MOBA	League of Legends	PC	KBM	Team
Sports	Rocket League	PC/Console	GP/KBM	Team
Sports	FIFA	Console/PC	GP/KBM	Single
FPS	Counter Strike	PC	KBM	Team

FPS -First person shooter; RTS–Real Time Strategy; MOBA–Multiplayer Online Battle Arena; FPS–First Person Shooter; KBM–Keyboard and Mouse; GP–Gamepad.

Counter Strike (CS) is the most popular esport in the action or first-person shooter genre; the game is played in teams (5 vs 5). The principle of the game follows an attacking-defending paradigm: One team has to lay a bomb and protect it until it explodes, while the other team tries to prevent bomb laying and neutralize the bomb. The avatars are controlled from a first-person perspective and are equipped with various weapons, which may be used to eliminate enemies. Competitive games are mostly played in a best of 30 rounds mode, with a round duration of approximately 2 minutes. CS is played on the PC with mouse and keyboard (https://counterstrike.fandom.com/wiki/Competitive; last check on 10.12.2019).

Starcraft II (SCII) is a renowned single player (1 vs 1) real time strategy (RTS) esport. To win a game in Starcraft II, the players have to build and control different units from bird’s-eye view and destroy the enemies’ base. The game duration is not fixed (average: 10–15 minutes) but can vary considerably. Starcraft II is played exclusively on PC with mouse and keyboard.

League of Legends (LoL) is currently the most popular esport worldwide [2]. LoL as a “Multiplayer Online Battle Arena (MOBA)” game is played in teams (5 vs 5). The game includes elements of real time strategy games, i.e., the players have to destroy the enemies’ base. In contrast to RTS games, each player controls only one single character from the bird’s-eye view. The average game duration is approximately 28 minutes (https://www.leagueofgraphs.com/rankings/game-durations/na; last check on 10.12.2019). LoL is also played exclusively on PC with mouse and keyboard.

Rocket League (RL) is currently the most popular team esport in the sports genre. In competitive gaming, RL is mainly played in teams (3 vs 3) and is in principle a vehicular soccer game. The players control their cars from a first-person perspective and have to score goals. The specificity of the game is that the controlled cars are not only able to fly, but also to destroy opponent cars. The controls in RL are challenging, as the avatar (car) can be rotated around the longitudinal, as well as transverse and vertical axis by means of hand and finger movements. RL is played on PC as well as on consoles (X-Box One, Playstation 4, Nintendo Switch). The game duration is exactly 5 minutes, plus overtime added in case of a draw (https://www.rocketleagueesports.com/rules/; last check on 10.12.2019). The game can be played with keyboard and mouse or a gamepad, however, the use of a gamepad is more common.

FIFA is a soccer simulation and the most popular single player (1 vs 1) game in the sports genre. The game can be termed a “virtual simulation” of soccer. While FIFA can be played on PC, it is more commonly played on Consoles (Xbox One and Playstation 4) in esports tournaments. The game duration in competitive FIFA is 12 minutes (https://proplayers.eu/en/rules/fifa-online-tournament-rules; last check on 10.12.2019). The most used control device is the gamepad.

### Hypotheses (H) and research questions (RQ)

Based on the analysis of the current state of research, the following hypotheses and research questions were addressed in this study (Tables 6 and 7). H1 to H6 have been derived either from theory or from empirical results, whereas RQ1 to RQ6 address issues that have not yet been sufficiently addressed by theory and empirical studies.

**Table 6:** Hypotheses (H).

	Description
H1	The importance of specific competencies depends on the specific esport.
H2	The importance of specific training areas depends on the specific esport.
H3	Training engagement in specific training fields depends on the specific esport.
H4	A positive correlation between the motivation and training effort exists.
H5	A positive correlation between skill level and training engagement exists.
H6	A negative correlation between playing time and subjectively rated fitness level exists.

**Table 7:** Research questions (RQ).

	Description
RQ1	Is there a correlation between the skill level and playing time, playing time without a break and break duration?
RQ2	Is there a relation between the skill level and preferred training information sources?
RQ3	Is there a correlation between estimated importance of and actual training engagement in specific training areas?
RQ4	Do esports competencies show a specific factor structure?
RQ5/6	Does (desired/actual) esports training show a specific factor structure?

Based on the above-mentioned features and basic knowledge of the introduced games, initial assumptions with respect to the differential importance of single factors to esports performance were derived (Table 8). Factors not mentioned are expected not to differ between the games.

**Table 8:** Assumptions about differences between the esports.

	Coordination		Tactical-cognitive	Condition		Social
Game	Accuracy	Spatial orientation	Strategic thinking	Speed	Endurance	Teamwork/Team feedback
SCII	**	*	***	***	***	-
LoL	**	*	***	**	***	***
RL	***	***	**	**	**	***
FIFA	**	*	**	**	**	-
CS	***	**	***	**	**	***

CS = Counter Strike; SCII = Starcraft II, LoL = League of Legends, RL = Rocket League.

*** important or very important;

**somewhat important;

* rather unimportant;— = not important at all.

Accuracy is expected to have a higher rating of importance for RL and CS players than SCII, LoL and FIFA players, since aiming is described as one of the basic mechanics of CS [34]. To control the avatar (car) in RL, very accurate and fine movements are mandatory.

Spatial orientation is likely to be considered particularly important by RL players, because of the challenging game mechanics and a great number of three-dimensional movement possibilities. In CS, spatial orientation also plays an important role, as the players play from the first person's perspective and orient themselves precisely in the given 3D space (map). In SCII, LoL and FIFA, the player has an overview from a bird's eye view. For this reason, the spatial orientation in these esports might not play as big of a role as in CS and RL.

Strategic thinking is expected to be more important in tactics and strategy esports games (CS, SCII, LoL). However, tactics can be decisive in any esport and are therefore important in FIFA and RL as well.

The action speed is considered important in all studied esports. Nevertheless, it can be expected that skills related to action speed will be considered particularly important by SCII players, as frequent and extremely fast movements are mandatory [46].

The endurance to perform over a longer period of time is important in every esport. Tournaments are often performed on one single day. The players have to maintain top level of their performance all day long during the tournament. However, in some esports endurance is particularly important. In SCII and LoL, games can have a much longer duration. The players have to maintain their concentration over a longer period of time. In CS, RL and FIFA the individual games are shorter, which gives the players the opportunity to recover.

Reasonably, teamwork may be more important in multiplayer disciplines (CS, LoL, RL) as compared to single-player disciplines (SCII, FIFA).

### Survey

To reach a great number of esports players, an online survey method was applied in this study. For the creation and implementation of the survey, the platform “sosci-survey” was used. Closed single- and multiple-choice questions as well as scale and valued measure questions were developed, while some questions were a mixture of open and closed questions (Table 9). In total, 14 questions were administered. The complete questionnaire can be viewed in the S1 File.

**Table 9:** Questions and scales applied in the survey.

Part	Questions	Scale
General	Esports game selection, 5 items	Single Choice
	Skill level, 6 items	Single Choice
Motivation	6 designated statements, 1 open statement	5-pt. Likert: 1—Does not apply at all– 5 fully applies
Competencies relevant to esports	19 items	5-pt. Likert 1 –not important at all– 5 very important
Training behaviour	Playing time	Format: hours–xxx.x (range: 0.5–168)
	Training time	Percent of playing time
	Playing time until break	Format: hours–xxx.x
	Break length	Format: minutes–xxx
Information source for training methods	5 designated statements, 1 open statement	Multiple choice
Training areas	Training area Importance, 8 items	5-pt. Likert 1 –not important at all– 5 very important
	Training frequency, 8 items	5-pt. Likert 1 –never; 5- very often
Demographic/Fitness	Fitness-level	5-pt.: 1 –very low; 5 -very high
	Age	Integer number
	Gender	Male, female, diverse

For the publication of the survey, the online platforms Reddit, Facebook and Discord were used. Reddit is one of the most visited sites on the internet (https://www.alexa.com/siteinfo/reddit.com; last check on 10.12.2019) and is very popular in the esports community. Every relevant esports game has its own subforum. Only the FIFA subforum did not allow to publish the survey. Therefore, public Facebook groups and the communication platform “discord” were used to recruit FIFA players (Table 10).

**Table 10:** Forum members for the esport games selected for this study.

Game	Platform	Forum members*
Starcraft II	Reddit	~220,000
Rocket League	Reddit	~486,000
League of Legends	Reddit	~2,530,000
Counter Strike	Reddit	~810,000
FIFA	Facebook, Discord	~44,000

* Reference date: 17.03.2019.

Before the publication, a pretest with 8 selected individuals was conducted to check the completion time and improve the comprehensibility of the survey. A completion time of 5 to 8 minutes was determined. After the pretests, 3 items were modified to improve the comprehensibility.

### Ethical issues

According to the guidelines of the German Research Association (https://www.dfg.de/en/research_funding/index.html; last check on 10.12.2019), an approval by the Ethics Committee was not required, as the study did not involve medical treatment nor exposure of the participants to physical or psychological risk. This position was confirmed informally by two members of the Ethics committee.

In this study, we invited members and visitors in open forums for voluntary participation. Due to the large number of entrants and the fact, that we did not record any personal information that could be used to identify the participants (i.e. names, e-mails or IP-addresses), anonymity was preserved.

### Statistical analysis

For the data analysis, IBM SPSS Version 25 was used. The statistical tests are specified in Table 11

**Table 11:** Statistical analysis methods related to the hypotheses and research questions.

	Method
H1-H3	Repeated-measures two-factor ANOVA: • Factor 1 –game (5) • Factor 2 –measure
	Follow-up tests: Mann-Whitney U-tests
H4-H6, RQ3	Pearson correlation
RQ1	Spearman rank correlation
RQ2	χ^2^ test and Phi coefficient
RQ4 –RQ6	Factor analysis (main components), Varimax rotation, KMO, Eigenvalues ≥ 1; Scree plots

## Results

### Sample

**Fig 7:**
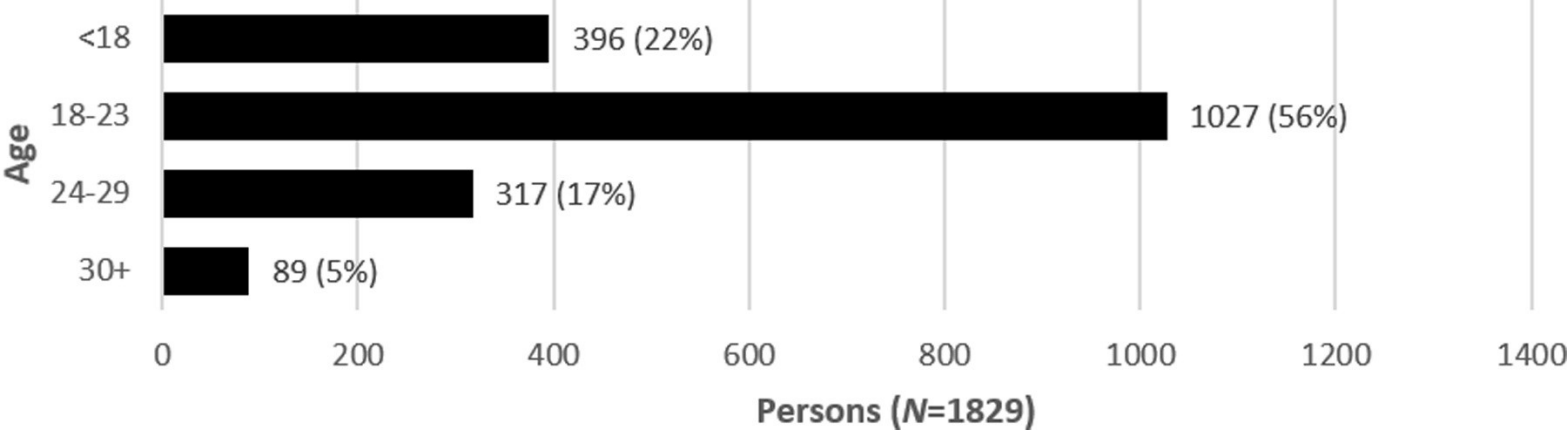
Age distribution.

Overall, 1,835 participants completed the survey. Table 12 shows the distribution and response rates regarding the five esports. Participants’ age ranged from 13 to 47 years (M = 20.9; SD = 4.5; Fig 7).

**Table 12:** Sample distribution.

Game	Surveys completed	Forum members*	Response rate
Starcraft II	124 (7%)	~220,000	0.06%
Rocket League	598 (33%)	~486,000	0.12%
League of Legends	128 (7%)	~2,530,000	0.01%
Counter Strike	927 (50%)	~810,000	0.11%
FIFA	58 (3%)	~44,000	0.13%
**Total**	**1835**		

* Reference date: 17.03.2019.

**Fig 8:**
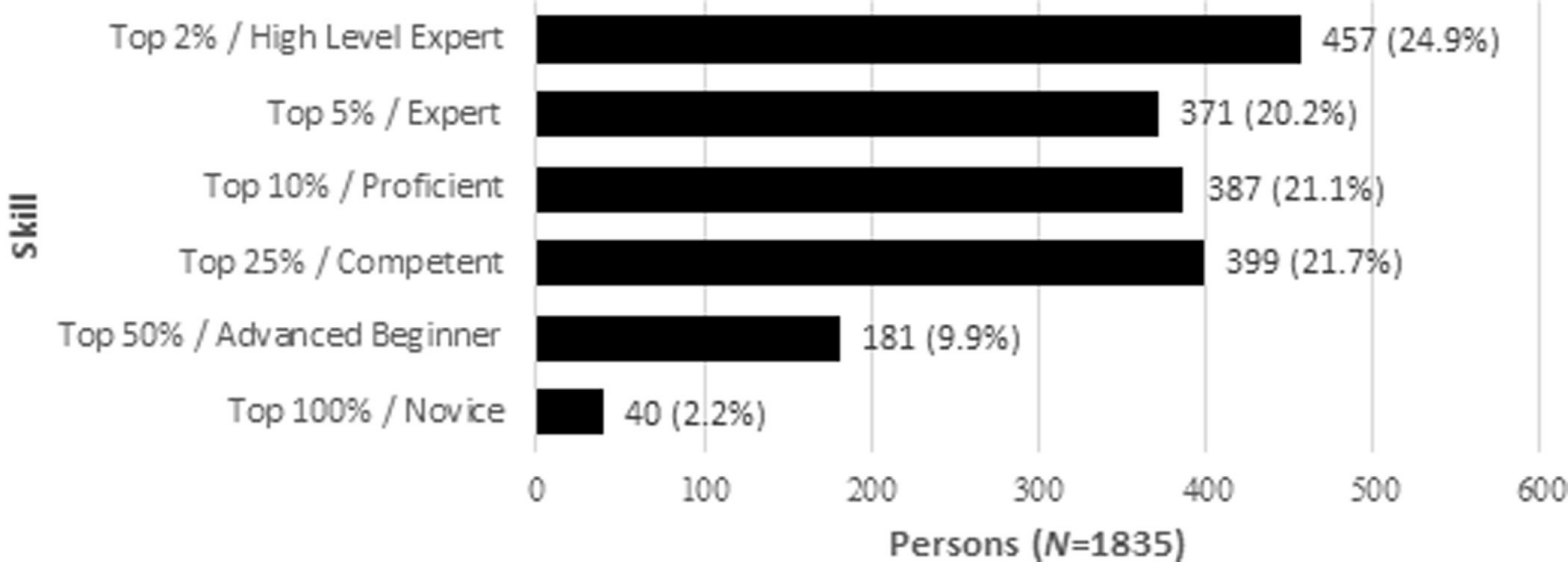
Skill level distribution.

The gender of the participants is dominated by males (*N* = 1,749; 95%). Furthermore, 67 (4%) females and 11 (1%) diverse took part in the survey. The skill level of the participants was rated high on average, i.e., mainly experienced players took part in the survey (Fig 8). 457 out of 1,835 respondents were in the top 2%, while 371 players were in the top 5% and 387 in the top 10% of the respective esports player base.

The total playing time of 20.03 hours (SD = 15.8) per week was reported by the participants. The relative training time was 38.85% of the total playing time. Therefore, the respondents exercise for 7.78 hours per week.

### General findings

**Fig 9:**
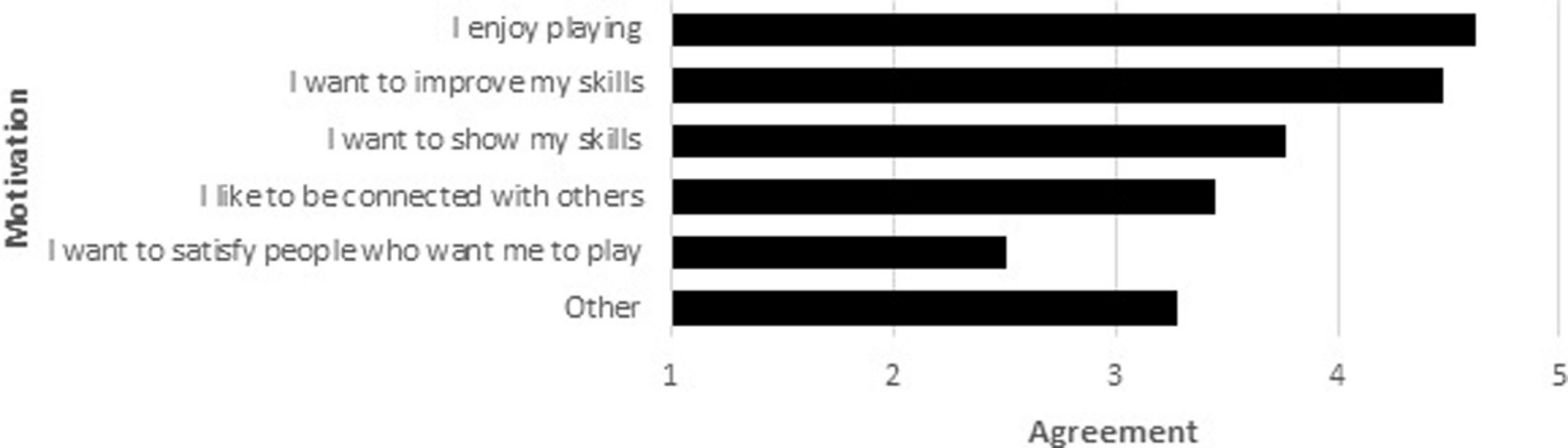
Motivation to play esports games. Scale: 1 –Does not apply at all, 3 –somewhat applies, 5 –fully applies.

Fig 9 shows that the enjoyment of playing and the motivation to improve skills are the main reasons to engage in esports, followed by showing off skills and connection to others. Satisfying other people is the least important aspect.

**Fig 10:**
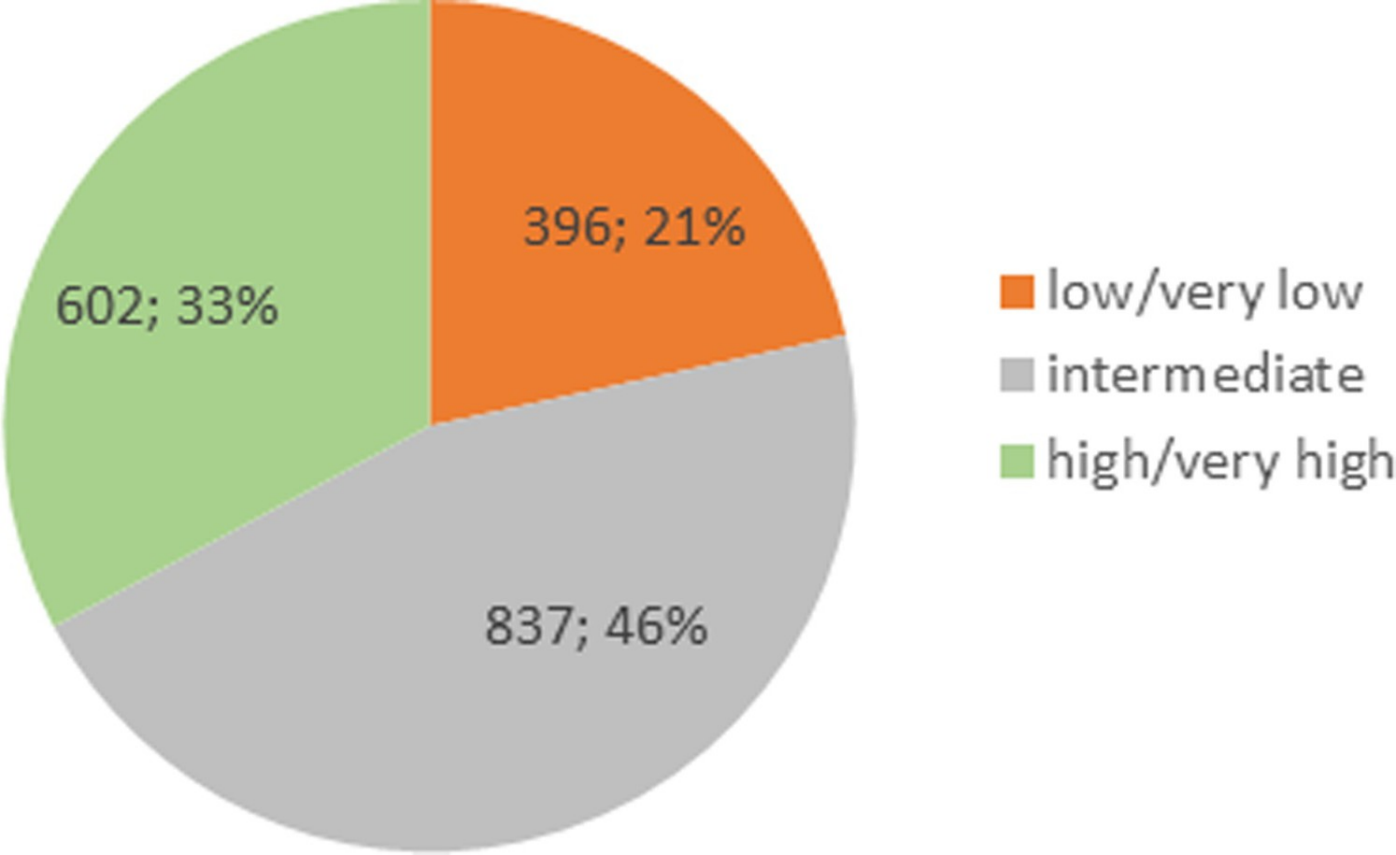
Estimated fitness level. (*N* = 1835).

Regarding fitness level, most players (46%) estimated their fitness level as intermediate, whereas 33% rated their fitness level high or very high and 21% rated it low or very low (Fig 10).

**Fig 11:**
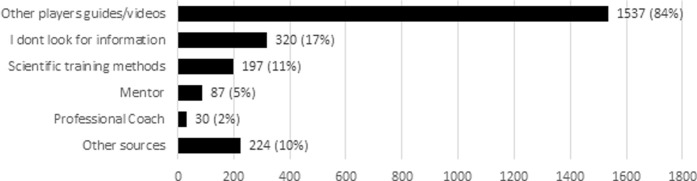
Training method sources. (*N* = 1835).

The players get information for their training methods from different sources (Fig 11). Very few players have a professional coach (2%) or mentor (5%). Most players use other players as a source for training methods (84%), whereas 17% of the players stated that they do not specifically search for information on training methods and 10% use other sources.

### Hypothesis and research question testing

#### H1 –the importance of specific competencies depends on the specific esport

**Fig 12:**
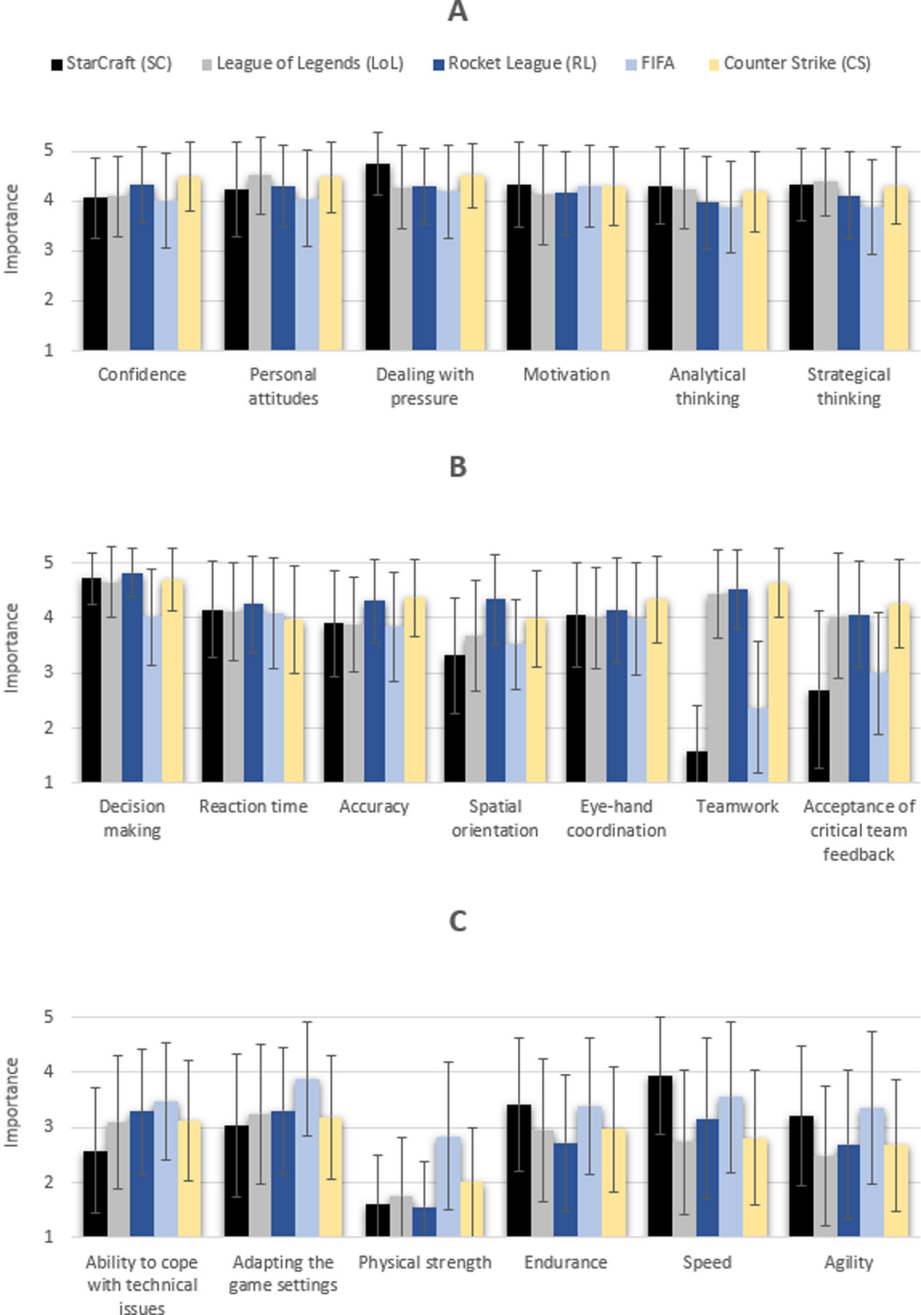
Importance of 19 competencies (means and SD). Scale: 1 –not important at all; 3 –somewhat important; 5 –very important.

Fig 12 shows that the importance of competencies varies in different esports (for means and standard deviations, see S1–S3 Tables). In SCII, dealing with pressure and decision making are particularly important, while team-related competencies and physical strength are considered least important. In LoL, personal attitudes, strategic thinking, decision-making and teamwork were rated as most important, while speed, agility and physical strength were found to be less decisive. In RL and CS, the competencies of personal attitude, confidence, decision making, accuracy, spatial orientation and teamwork dominate. In contrast, the competencies of physical strength, agility and speed were rated less important. In FIFA, motivation and reaction time are the most crucial competencies, while physical strength is the least important.

Regarding competencies, game-dependent profiles were identified. Confidence, accuracy, spatial orientation and hand-eye coordination were considered more important in RL and CS than in the other three e-sports. The competence of dealing with pressure was of paramount importance for CS and SCII. Analytical thinking, strategic thinking, decision-making and personal attitude were considered less important in FIFA than in SCII, LoL, RL, and CS. In terms of motivation and response time, a similar assessment of significance was found for all e-sports studied, while teamwork and acceptance of team feedback were rated much more important by LoL, RL and CS than by SCII and FIFA players. The handling of technical problems, the adaptation of game settings and physical strength showed a greater significance in FIFA compared to the other e-sports examined. The importance of endurance, speed and agility was rated highest by the SCII and FIFA players.

A two-factor ANOVA revealed significant main effects of e-sports disciplines and competence as well as a significant interaction (Table 13).

**Table 13:** Results of the two-factor ANOVA regarding competence fields in different esports.

Factor	df1	df2	F	p	n²
Competence	11	19783	433.431	<0.001	.191
Game	4	1830	12.895	<0.001	.027
Competence x Esport	43	19783	47.68	<0.001	0.094

Mann-Whitney U follow-up tests (S4 and S5 Tables) show significant differences between the esports games (Table 14). The most significant differences were found between RL and CS, as well as CS and FIFA (17). The fewest significant differences were found between LoL and CS (7). Regarding the competencies, the most differences were found for teamwork (9), speed (8), and spatial orientation (8), while the least significant differences were discovered in reaction time (1) and motivation (2). Overall, at least one significant difference between the e-sports disciplines was found for each competence. Therefore, H1 is confirmed.

**Table 14:** Significant differences between the esports in the importance of specific training areas (LoL = League of Legends; RL = Rocket League; CS = Counter Strike; D = Differences).

Training area	Starcraft II				League of Legends			Rocket League		FIFA	D
	LoL	RL	FIFA	CS	RL	FIFA	CS	FIFA	CS	CS	
Confidence		x		x	x		x	x	x	x	7
Personal attitudes	x			x	x	x		x	x	x	7
Dealing with pressure	x	x	x	x			x		x	x	7
Motivation		x							x		2
Analytical thinking		x	x		x	x			x	x	6
Strategic thinking		x	x		x	x			x	x	6
Decision making		x	x		x	x		x	x	x	7
Reaction time									x		1
Accuracy		x		x	x		x	x		x	6
Spatial orientation	x	x		x	x		x	x	x	x	8
Eye-hand coordination				x			x		x	x	4
Teamwork	x	x	x	x		x	x	x	x	x	9
Acceptance of critics	x	x		x		x		x	x	x	7
coping w. technical issues	x	x	x	x		x			x	x	7
Adapting the game settings		x	x			x		x	x	x	6
Physical strength			x	x		x	x	x	x	x	7
Endurance	x	x		x		x		x	x	x	7
Speed	x	x		x	x	x		x	x	x	8
Agility	x	x		x		x		x		x	6
Differences	9	15	8	13	8	12	7	12	17	17	

#### H2 –the importance of specific training areas depends on the specific esport

**Fig 13:**
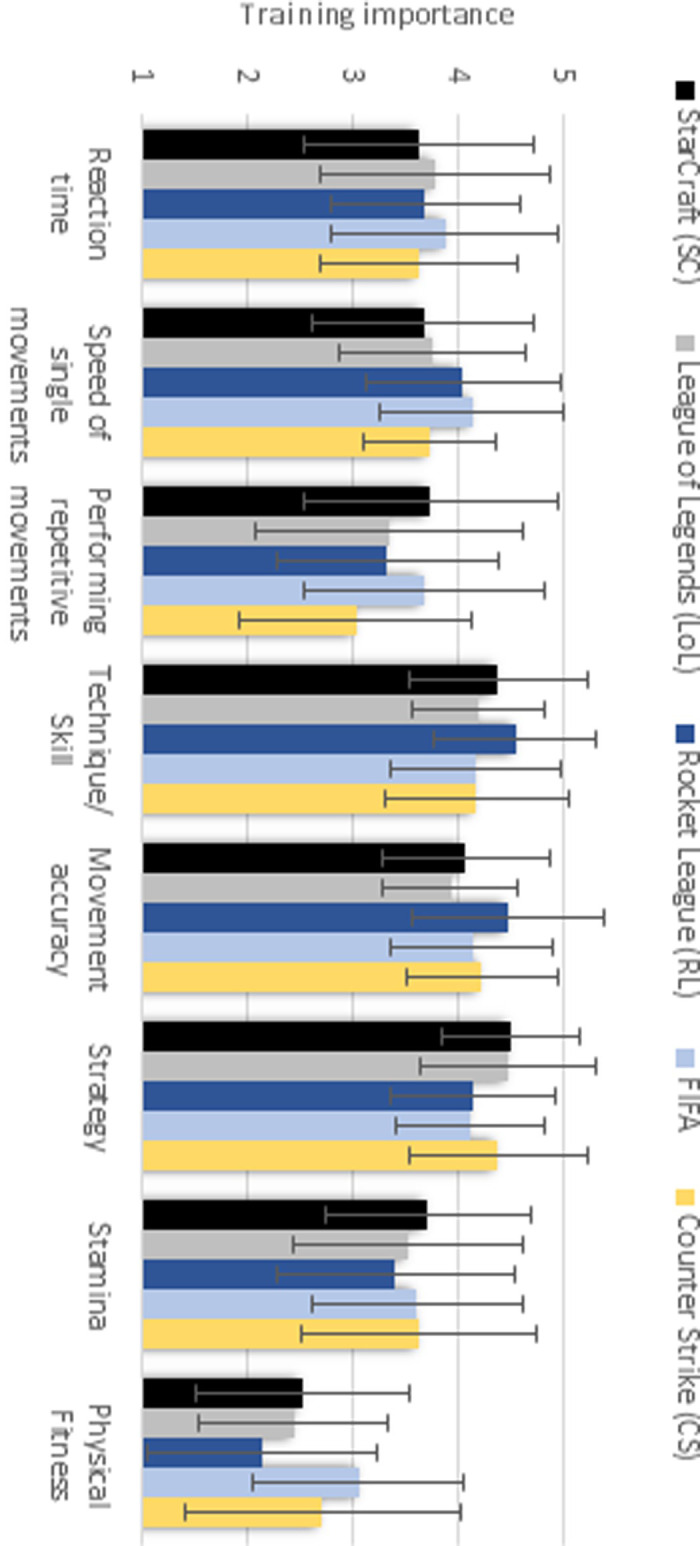
Importance of training fields. Scale: 1 –not important at all; 3 –somewhat important; 5 –very important.

In Fig 13 the importance of specific training areas is illustrated (for means and standard deviations, see S6 Table). All studied esports identified technique, movement accuracy and strategy as the most important training areas. In RL and FIFA, speed of single movements was rated essential as well. For every esport, physical fitness was rated the least important training area.

The training areas of reaction time and stamina were rated similarly important by all studied esports. Training of speed of single movements was considered most relevant in RL and FIFA, while training of repetitive movements was considered most critical in SCII and FIFA. Both, technique training and movement accuracy training were rated most important by RL players. Strategy training was considered most important by SCII, LoL and CS players. The importance of physical fitness training was rated highest by FIFA players.

A two-factor ANOVA revealed a significant main effect of training area as well as a significant interaction of esport and training area/field (Table 15).

**Table 15:** Results of the two-factor ANOVA regarding training field importance in different esports.

Factor	df1	df2	F	p	n²
Area (Importance)	6	10944	330.962	<0.001	0.153
esport	4	1830	2.068	0.083	0.004
Area x Esport	24	10944	21.186	<0.001	0.044

Mann-Whitney U follow-up tests (see S7 and S8 Tables) show significant differences between the esports (Table 16). The most significant differences were found between RL and CS (7) and SCII and RL (6). The least significant differences were found between SCII and LoL (2). From the perspective of the training areas, most significant differences were found in the importance of training of physical fitness (8), strategy (7) and performing repetitive movements (7). The least differences were found regarding the importance of training reaction time, as no significant differences were found. Therefore, with the exception of reaction time, H2 was confirmed.

**Table 16:** Significant differences between the esports in the importance of specific training areas (LoL = League of Legends; RL = Rocket League; CS = Counter Strike; D = Differences).

Training area	Starcraft II				League of Legends			Rocket League		FIFA	D
	LoL	RL	FIFA	CS	RL	FIFA	CS	FIFA	CS	CS	
Reaction time											0
Single movements		x	x		x	x			x	x	6
Repetitive movements	x	x		x		x	x		x	x	7
Technique/skills	x			x	x			x	x		5
Movement accuracy		x		x	x		x	x	x		6
Strategy/tactics		x			x	x	x		x	x	6
Stamina		x							x		2
Physical fitness		x			x	x	x	x	x	x	7
Differences	2	6	1	3	5	4	4	3	7	4	

#### H3 –training engagement in specific training fields depends on the specific esport

**Fig 14:**
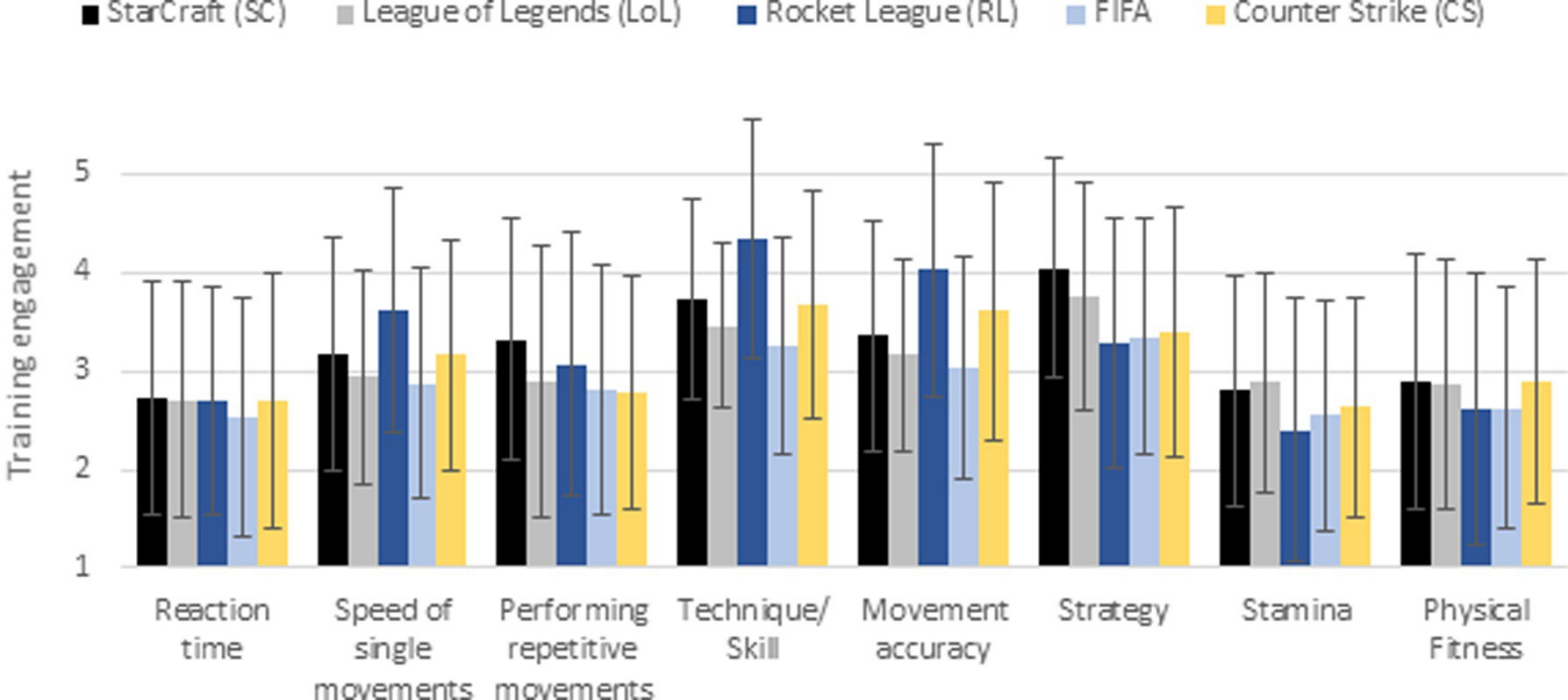
Engagement in training fields. Scale: 1 –never; 3 –occasionally; 5 –very often.

In Fig 14 the frequency of training in specific areas is illustrated (for means and standard deviations, see S9 Table). The most frequently trained areas of SCII players are strategy and technique. However, movement accuracy, speed of single movements and repetitive movements training seem to be relevant as well. The reaction time and stamina, on the other hand, are the least trained. LoL and CS players, most frequently train strategy, technique and movement accuracy. While reaction time receives the least attention for LoL players, CS players engage the least in stamina training. In RL a high priority is given to the training of technique and movement accuracy. While strategy and speed of single movements are also among the relevant training areas, stamina and physical fitness are the least frequently trained areas in RL. FIFA players most frequently train strategy and technique, while reaction time, stamina and physical fitness get the least attention.

Regarding the training areas, specific profiles were identified. Speed of single movements, as well as technique and movement accuracy are most often trained by RL players, while FIFA players are least likely to practice these areas. Stamina and physical fitness training are performed more often by SCII, LoL and CS players than by RL and FIFA players. Repetitive movements are most often trained by SCII and RL players, while strategy training is most frequently addressed by SCII and LoL players. The training frequency of reaction time was similar in all esports.

A two-factor variance analysis revealed significant main effects of training area and esport as well as a significant interaction (Table 17).

**Table 17:** Results of the two-factor ANOVA regarding training field engagement in different esports.

Factor	df1	df2	F	p	n²
Area (Frequency)	6	10658	120.508	<0.001	0.062
Game	4	1830	7.066	<0.001	0.015
Area x Esport	23	10658	20.039	<0.001	0.042

Mann-Whitney U follow-up tests (see S10 and S11 Tables) show significant differences between each esport (Table 18). The most significant differences regarding the training frequency of specific training areas were found between SCII and RL (6) and RL and CS (6). The least significant differences were discovered between SCII and LoL (1) and LoL and FIFA (1). Regarding the specific training areas, most differences were found in the training frequency of movement accuracy (7). The fewest differences were found in the training frequency of reaction time (0) and physical fitness (2). Therefore, with the exception of reaction training, H3 could be confirmed.

**Table 18:** Significant differences between the esports in the frequency of training in specific training areas (LoL = League of Legends; RL = Rocket League; CS = Counter Strike; D = Differences).

Training area	Starcraft II				League of Legends			Rocket League		FIFA	D
	LoL	RL	FIFA	CS	RL	FIFA	CS	FIFA	CS	CS	
Reaction time											0
Single movements		x			x		x	x	x		5
Repetitive movements	x		x	x					x		4
Technique/skills		x	x		x			x	x	x	6
Movement accuracy		x		x	x		x	x	x	x	7
Strategy/tactics		x	x	x	x	x	x				6
Stamina		x			x		x		x		4
Physical fitness		x							x		2
Differences	1	6	3	3	5	1	4	3	6	2	

#### H4 –a positive correlation between the motivation and training effort exists

Significant positive correlations between showing and improving skills and training effort were revealed. A significant, but weak negative correlation was found between enjoyment and training effort. No correlations were discovered between social connectness and social satisfaction and training effort (Table 19).

**Table 19:** Pearson correlation coefficients regarding the relationship of motives and training time.

Correlation: Training time with …		
Item	N	r
I enjoy playing	1813	-0.049*
I want to show my skills	1824	0.139**
I want to improve my skills	1827	0.246**
I like to be connected with others	1818	-0.046
I want to satisfy people who want me to play	1807	0.028

* 2p < .05;

** 2p < .001.

In sum, H4 could not be fully confirmed. Rather selected aspects of motivation are related to training effort.

#### H5 –a positive correlation between skill level and training engagement exists

A significant, but weak correlation of skill level and training engagement was found (N = 1,835; r = 0.153, 2p<0.001).

#### H6 –a negative correlation between playing time and subjectively rated fitness level exists

A significant, but weak negative correlation between the playing time and the subjectively assessed fitness level was found (*N* = 1835; r = -0.056, 2p<0.05).

#### RQ1 –is there a positive correlation between the skill level and playing time, playing time without a break and break duration?

A significant positive weak correlation was found between the subjective ability level and the playing time as well as playing hours until the break. A negligible correlation was found between the skill level and the length of the breaks (Table 20).

**Table 20:** Spearman rank correlation between skill level and playing time, pause length and playing time until break.

		Skill Level	
	Playing time	Pause length	Playing time until break^#^
Pearson Correlation (r)	0.288**	0.050*	0.112**
Significance (2-tailed)	<0.001	0.032	<0.001
N	1835	1835	1833

^#^ 2 outliers have been eliminated;

* 2p < .05;

** 2p < .001.

#### RQ2 –is there a correlation between the skill level and preferred training information sources?

Significant but weak relations between skill level and the use of a professional trainer, scientific training methods, other players as a source of information, but also with no search for information sources were discovered. No statistically significant relation was found between the skill level and the mentor as an information source (Table 21).

**Table 21:** Relation between skill level and professional coach, mentor, scientific training methods, other players and no information search.

Relation between skill level and…	χ^2^	df	Asymptotic significance (two-sided)	Phi	Asymptotic significance
Professional coach	23.122^a^	5	<0.001	0.112	<0.001
Mentor	6.972^a^	5	0.223	0.062	0.223
Scientific training methods	20.524^a^	5	0.001	0.106	0.001
Other players (guides, videos, etc.)	31.706^a^	5	<0.001	0.131	<0.001
No information	17.199^a^	5	0.004	0.097	0.004
Number of valid cases	1835				

#### RQ3 –is there a positive correlation between estimated importance and actual training engagement regarding specific training areas?

In general, a moderate to weak relationship of attitude and behavior was revealed. The strongest correlations were found in reaction time and repetitive movements, while the weakest correlations were found in physical fitness (Table 22).

**Table 22:** Correlation between actual engagement and estimated importance of training areas.

Training area (estimated–actual)	N	Correlation (r)
Physical fitness	1835	.327**
Tactics/strategy	1835	.425**
Skills	1835	.427**
Speed	1835	.438**
Accuracy	1835	.444**
Stamina	1835	.476**
Reaction time	1835	.513**
Repetitive movements	1835	.632**

* 2p < .05;

** 2p < .001.

#### RQ4 –the factor structure of esports competencies

An exploratory factor analysis (N = 1835; KMO = 0.79) revealed a six-factor solution explaining 62% of variance (Table 23). All 19 variables showed a substantial loading on just one factor.

**Table 23:** Factor analysis–competence structure (only factor loadings > 0.5 are displayed).

	Component					
	1	2	3	4	5	6
**Explained variance [%]**	**13.7**	**11.2**	**10.2**	**9.4**	**9.0**	**8.6**
**Confidence**				0.663		
**Personal attitudes**				0.634		
**Dealing with pressure**				0.677		
**Motivation**				0.546		
**Analytical thinking**			0.805			
**Strategic thinking**			0.834			
**Decision making**			0.641			
**Reaction time**		0.718				
**Accuracy**		0.768				
**Spatial orientation**		0.602				
**Eye-hand coordination**		0.673				
**Teamwork**					0.855	
**Acceptance of critical team feedback**					0.765	
**Ability to cope with technical issues**						0.811
**Adapting the game settings**						0.815
**Physical Strength**	0.637					
**Endurance**	0.757					
**Speed**	0.838					
**Agility**	0.871					

The six components can be interpreted as follows:

- Component 1: physical competencies (condition)
- Component 2: sensori-motor or coordinative competencies
- Component 3: strategic-cognitive competencies
- Component 4: psychic competencies
- Component 5: social competencies
- Component 6: media-related competencies

#### RQ5 –structure of critical training components

Regarding components of training that were estimated important by the esports athletes, factor analysis (N = 1835; KMO = 0.71) revealed a three-factor solution with a clear contribution of the 8 variables explaining 60.5% variance (Table 24):

- Component 1: speed
- Component 2: strategy and psychophysical fitness
- Component 3: sensori-motor control

**Table 24:** Estimated training structure.

	Component		
	1	2	3
**Explained variance [%]**	**22.2**	**19.4**	**18.9**
**Reaction time**	0.729		
**Speed of certain single movements**	0.753		
**Performing repetitive movements as fast as possible**	0.668		
**Technique/Skills**			0.813
**Movement accuracy**			0.810
**Strategy/Tactics**		0.675	
**Stamina**		0.721	
**Physical fitness**		0.727	

The identified factors closely resemble the first three factors found in the estimated competence structure.

#### RQ6 –structure of actual training

Regarding actual training of the esports athletes, a factor analysis (N = 1,835; KMO = 0.78) revealed a two-factor solution explaining 54.5% variance (Table 25):

- Component 1: speed and sensori-motor control
- Component 2: strategy and psychophysical fitness

**Table 25:** Actual training structure.

	Component	
	1	2
**Explained variance [%]**	**33.6**	**20.9**
**Reaction time**	0.523	
**Speed of certain single movements**	0.808	
**Performing repetitive movements as fast as possible**	0.691	
**Technique/Skills**	0.768	
**Movement accuracy**	0.724	
**Strategy/Tactics**		0.588
**Stamina**		0.777
**Physical fitness**		0.741

Therefore, a distinction between speed of actions and accurate sensori-motor control is not present anymore: Speed and accuracy are collapsed in actual training.

## Discussion

In the following, the results are discussed and an outlook on further research is given. The results of the hypotheses and research questions are summarized in Table 26.

**Table 26:** Summary of results.

Hypothesis & Question	Result
H1	confirmed
H2	confirmed with the exception of reaction time
H3	confirmed with the exception of reaction time
H4	partly confirmed
H5-H6	confirmed
RQ1	weak positive correlations between skill level and playing time and playing time until break
RQ2	significant relations between skill level and information sources were found with the exception of mentor
RQ3	moderate to weak relationship between attitude and behavior
RQ4	six factor solution (representing the integrative model)
RQ5	three factor solution
RQ6	two factor solution

### Sample

Only a small proportion of the forum members was recruited for the study (0.06–0.13%; see Table 12). It must be mentioned that the public accessibility of the forums offers the possibility of consuming the forum content without registering, which is why the recruitment proportion of the total invited persons is to be estimated even lower.

Participants’ age ranged from 13 to 47 years (M = 20.9; SD = 4.5), while the participants gender was dominated by males (95%). The results are conforming to the study of Adamus [48], where the average age was found out to be 20.01 (SD = 4.02) and 97% of the respondents were males.

Players spend an average of 20.03 (SD = 15.8) hours a week playing, but only 38.85% (7.78 h/wk) of the playing time is used for training. In the study of Adamus [48], players trained 14 hours a week on average, whereas Kari and Karhulahti [49] found a training time of approximately 37 hours a week. In comparison to our study, training times are much higher. The weekly playing time, on the other hand, is comparable to the average weekly playing time of hobby level players (21.8 h/wk) in the study of Froboese et al [6].

Since there was an imbalance between the participants of the different esports and a dominant representation of CS (50%) and RL (33%) compared to the other esports (FIFA 3%; SCII 7%; LoL 7%), a different degree of representativity can be expected for every esport involved in this study.

### H1 –the importance of specific competencies depends on the specific esport

The results of H1 revealed that the esports addressed in this study differ in the estimated importance of competencies. At least one significant difference between the esports was found for every competence.

Before the survey, assumptions were made regarding the importance of individual competencies. Differences in the esports were expected in the competencies of accuracy, spatial orientation, strategic and analytical thinking, action speed, endurance and team related competencies such as teamwork and acceptance of critical team feedback (Table 8).

In the upper part (coloured) of Table 27 the results of the importance assessment from H1 are summarized on the basis of the evaluation pattern from Table 8:

**Table 27:** Results regarding the differences between the esports in competence importance estimation (Green = Confirmed assumption; Red = non-confirmed assumption).

Competence			Game		
	SCII	LoL	RL	FiFA	CS
Accuracy	**	**	***	**	***
Spatial Orientation	** [*]	** [*]	***	** [*]	**
Strategic thinking	***	***	***[**]	**	***
Analytical thinking	***	***	**	**	***
Action speed	** [***]	* [**]	**	**	* [**]
Endurance	** [***]	* [***]	* [**]	**	* [**]
Teamwork	-	***	***	* [–]	***
Acceptance of critical team feedback	* [–]	***	***	** [–]	***
Confidence	***	***	***	***	***
Personal attitudes	***	***	***	***	***
Dealing with pressure	***	***	***	***	***
Motivation	***	***	***	***	***
Decision making	***	***	***	***	***
Reaction time	***	***	***	***	**
Eye-hand coordination	***	***	***	**	***
Ability to cope with technical issues	*	**	**	**	**
adapting the game settings	**	**	**	**	**
physical strength	-	-	-	*	*
Agility	**	*	*	**	*

1.00–1.99 = -; 2.00–2.99 *; 3.00–3.99 **; ≥4 ***.

*** important or very important;

**somewhat important;

* rather unimportant;— = not important at all.

In terms of accuracy, the predicted profile was consistent with the one found in the study. As expected, accuracy was significantly more important in the esports RL and CS than in the other esports. This is mainly due to the game mechanics in the respective games. In CS, the accuracy of aiming is one of the basic mechanics [34]. In RL, accurate movements are also very important in order to optimally align the avatar with the complex controls in order to score goals. In the esports SCII, LoL and FIFA, accurate movements are somewhat important as well, but are not as decisive as in RL and CS.

With regard to spatial orientation, the expected great importance in RL was confirmed. The control in RL is particularly challenging because the avatar (car) can fly and be controlled around all three axes. An additional difficulty in RL is the "ball cam". The "Ball cam" is an indispensable camera setting for the players, that always aligns the third person view of the player towards the ball. The associated fast changes of direction render the orientation much more difficult. These complex controls pose high requirements on the orientation. As expected for CS, spatial orientation fell into the group of "somewhat important" competencies. In CS, the avatar is controlled from the first person's perspective, which also poses challenges for orientation due to the limited field of vision but does not have as much freedom of movement and orientation requirements as the controls in RL. In SCII, LoL and FIFA the importance of spatial orientation was underestimated. However, estimations (between 3.31 and 3.67) are clearly below CS (3.99).

In the category of strategic and analytical thinking, the results only matched the predicted profile for analytical thinking. Strategic thinking had a greater importance in RL than expected. It is surprising that the estimated importance of strategic thinking in RL falls into the same category as in SCII, LoL and CS, since SCII and LoL are classified as strategy games. For CS, a tactical shooter, strategy is also a key element. RL, on the other hand, is a very fast game in which strategic thinking seems to be more important than expected.

For action speed, only two predictions were confirmed, i.e., RL and FIFA. In the remaining three games (SCII, LoL, and CS), action speed was overestimated. Since the fast execution of movements in SCII, LoL and CS is necessary in certain situations, this result is quite surprising.

With the exception of FIFA, endurance was overestimated. The lower importance of endurance, contrary to expectations, can be explained by the fact that esports players of all skill levels were included in the survey and therefore a regular tournament participation cannot be assumed for every player. Since tournaments in esports often take place on one single day, stamina most likely plays a greater role for regular tournament participants than hobby players.

In terms of teamwork and acceptance of team feedback, the predicted difference between team and individual esports was confirmed but overestimated. In LoL, RL and CS, the two competencies were rated more important than in the other two esports. It is surprising that acceptance of team feedback was estimated as “somewhat important” in FIFA, since this game is a single player esport.

Regarding confidence, personal attitudes, dealing with pressure, motivation and decision making, despite statistical differences, a great importance is documented for all games. Considering the means, the most important competence was decision making. Being able to make right decisions in fractions of a second is critical in all esports.

Reaction time represents the competence where the fewest differences between esports were discovered. The only significant difference was found between RL (4.26) and CS (3.97). CS was the only esport that did not rate reaction time as "important or very important". The significant difference in the importance of reaction time between RL and CS is very surprising, as quick reactions to the opponent or game action should play a similarly important role in both esports. Why this difference exists is therefore not clear and requires further investigation.

The eye-hand coordination was rated as equally important for all esports, with the exception of FIFA (3.98).

With one exception, media-related competencies were estimated as “somewhat important” for all esports. Only SCII players rated the ability to cope with technical issues as "rather unimportant”.

Physical strength was rated as less important in all esports, with small differences between the cluster of SCII, LoL and RL compared to FIFA and CS. Obviously, physical strength plays a less important role in the esports.

Agility was rated differently by the esports players as well. SCII and FIFA players rated this competence on average as "somewhat important" while the other three esports rated it on average as "rather unimportant". The differences between the esports can be explained by the fact that the mobility of the fingers could play a greater role in SCII and FIFA, since more keys are involved and a more frequent hitting with the fingers is necessary, than in the other esports. To confirm this thesis, however, further research will have to be carried out.

### H2 –the importance of specific training areas depends on the specific esport

This study revealed esports-specific differences regarding the estimated importance of training areas (Table 28). Significant differences were found for every training area with the exception of reaction time. Reaction time was rated as “somewhat important” in all esports addressed in this study.

**Table 28:** Results regarding the differences between the esports in training area importance estimation.

Training area			Game		
	SCII	LoL	RL	FIFA	CS
Reaction time	**	**	**	**	**
Speed of single movements	**	**	***	***	**
Repetitive movements	**	**	**	**	**
Technique/Skill	***	***	***	***	***
Movement accuracy	***	**	***	***	***
Strategy	***	***	***	***	***
Stamina	**	**	**	**	**
Physical fitness	*	*	*	**	*

1.00–1.99 = -; 2.00–2.99 *; 3.00–3.99 **; ≥4 ***.

*** important or very important; **somewhat important; * rather unimportant;— = not important at all.

The training of the speed of single movements was rated as important/very important by RL and FIFA players, while the other three esports rated it as somewhat important. The significant difference found between RL (4.05) and CS (3.74) was surprising, because acting faster than the opponent can be viewed as a win condition in many situations in both esports.

The training of repetitive movements was rated as somewhat important for all esports studied. Nevertheless, significant differences between the esports were discovered. The biggest significant difference was found between SCII (3.75) and CS (3.04). The difference can be explained by the fact, that repetitive movements are particular important in SCII, while there are not many repetitive movements in CS.

The training of the technical skills was rated as important/very important by every esport studied. The biggest significant difference was found between RL (4.55) and CS (4.18), FIFA (4.17) and LoL (4.20). In RL skills training is significantly more important because of the complicated and versatile controls of the avatar. In FIFA, LoL and CS the same skills are also required but are not as important and therefore not as high of a priority in training.

LoL players rated movement accuracy training as somewhat important, while the other esports rated it as important or very important. Even though the rating of SCII, RL, FIFA and CS is placed in the same category, significant differences between the esports were found. The most surprising significant difference was found between RL (4.49)and CS (4.23), as movement accuracy is considered crucial in CS [35] and in theory should not be rated lower than in RL. Overall the results show that movement accuracy training has a high value in the studied esports.

Strategy training was rated as important/very important by every studied esport as well. Significant differences between the esports were still found. The most noticeable differences were found between esports that are from or related to the strategy genre (SCII, LoL and CS) and the sports simulations (RL and FIFA). Due to the fact that the game principle of the first mentioned esports is much more strategy-oriented than in the two sports simulations, the results are not surprising. Nevertheless, as the results show, tactics training is important in every esports studied.

Stamina training was rated as somewhat important by every esport studied and only few significant differences (SCII and RL, RL and CS) were found. While RL players rated stamina training the lowest, SCII players rated it the highest in comparison to the other esports. This can be explained by the fact that SCII players have to perform many fast and repetitive moves over a longer period of time.

With the exception of FIFA, physical fitness training was rated as „rather unimportant” by every esport studied. The biggest significant difference was found between FIFA (3.07) and RL (2.16). Why this difference exists, is not clear. It is remarkable that physical fitness was the least important training area for every esport studied.

### H3 –training engagement in specific training fields depends on the specific esport

Differences in training frequency of the training areas were discovered (Table 29). Analogous to H2, only for reaction time no significant differences were found. Reaction time training is trained „rarely” in all esports studied.

**Table 29:** Results regarding the differences between the esports in training area engagement.

Training area			Game		
	SCII	LoL	RL	FIFA	CS
Reaction time	*	*	*	*	*
Speed of single movements	**	*	**	*	**
Repetitive movements	**	*	**	*	*
Technique/Skill	**	**	***	**	**
Movement accuracy	**	**	***	**	**
Strategy	***	**	**	**	**
Stamina	*	*	*	*	*
Physical fitness	*	*	*	*	*

1.00–1.99 = -; 2.00–2.99 *; 3.00–3.99 **; ≥4 ***.

*** often or very often; **occasionally; *rarely;— = never.

Speed of single movements are trained occasionally in SCII, RL and CS players, while LoL and FIFA players practice this area rarely. The most surprising significant difference in the training frequency of speed of single movements was found between RL (3.63) and CS (3.17). Although individual movements are particularly important in RL, the speed of individual movements, such as fast and precise mouse movements, also play an important part in CS, since fast aiming is a crucial part of the game. Therefore, the results are surprising.

Repetitive movements are occasionally trained in SCII and RL, while LoL, FIFA and CS players train them rarely. Regarding significant differences, results conform to the results of H2, as the greatest significant difference was discovered between SCII (3.31) and CS (2.78). Frequent training of repetitive movements is important in SCII, while they are not necessarily a win condition in CS and therefore probably not the main objective of training.

Technique or skill is trained often/very often by RL players, while the other studied esports players train it occasionally. The biggest significant difference was found between RL (4.35) and FIFA (3.26) players. Technique or skill is the most important and most frequently trained area in RL.

Movement accuracy is trained often or very often by RL players, while the other esports players train this area occasionally. The predominant significant difference of RL (4.03) to CS (3.61) is particularly contraintuitive, because the movement accuracy is one of the most important and central traits in CS.

Strategy training is performed often or very often by SCII players. LoL, RL, FIFA and CS players occasionally engage in strategy training. The significant difference in strategy training between SCII (4.05) and CS (3.39) is surprising, since in both esports the players are very dependent on the right strategy or tactics to win the game. There is no logical explanation for the results.

Stamina is trained rarely by all esports studied. The significant differences in stamina training can be explained by the different game durations of the studied esports (see Selection of esports games for the study).

Physical fitness is trained rarely in all esports studied as well. Significant differences were found between SCII and RL as well as RL and CS. There is no clear explanation why the frequency of physical fitness training between the esports should differ.

### H4 –a positive correlation between the motivation and training effort exists

The correlations found in this study were either negligible or weak. It is reasonable that intrinsic motivation, i.e., improving skills, shows the strongest correlation.

### H5 –a positive correlation between skill level and training engagement exists

A significant positive but weak correlation was found between skill level and training engagement. This may be due to the fact that training engagement comprises only about 39% of the total playing time (see RQ2).

### H6 –a negative correlation between playing time and subjectively rated fitness level exists

A negligible negative correlation was found. The difference to the results found by Froboese et al. [6] may be due to different operationalisations; Froboese et al. assessed sedentary time, whereas we recorded total playing time.

### RQ1 –is there a positive correlation between the skill level and playing time, playing time without a break and break duration?

Significant weak positive correlations were found between the skill level and the total playing time as well as the playing hours until a break, while the correlation between skill level and the break length was negligible. These results are consistent with the well-known spacing effect in learning which claims a superiority of learning with breaks [50,51]. In particular, the study of Metalis [52] shows, that players who practised video games in intervals, i.e. took many breaks with only a short playing time between the breaks (distributed practice), improved their skills faster than players who practised without a break (massed practice).

### RQ2 –is there a correlation between the skill level and preferred training information sources?

Only weak correlations were found between the subjective skill level and the use of a professional trainer and scientific methods as a source of information for training methods used.

It is particularly noteworthy that relatively few players use professional trainers (2%). This can probably be explained by the fact that professional trainers have to be paid. Another noticeable aspect is the low proportion of players who use scientific sources for their training methods (11%). This may indicate a need for transformable scientific information on correct and effective training methods in esports.

Significant correlations were also found between the skill level and the other players as a source of information. This is the dominating source of information for the players (84%). Other players' information is free of charge and can be obtained through easy communication. In addition, this type of information retrieval requires little effort. Whereas top-level and low-level players use this source less often (75% to 77%), players ranging from advanced beginners to experts rely on this source more often (85% to 90%). This could be due to the fact that beginners are new and may not yet know any players, while the highest tier players do not consider other players as a valuable source of information.

Another significant correlation was found between the skill level and players who are not looking for any information. In total, 17% of the players said they were not looking for any information at all. There was a tendency for beginners (28%) and top players (22%) to look less for information. This could be due to the fact that these beginners try to find their way without any external information at the beginning and deal with the game themselves before searching for training methods, while a substantial portion of highest tier players might think they do not need any additional information.

### RQ3 –is there a correlation between estimated importance and actual training engagement regarding specific training areas?

For all training areas, significant correlations were found, with explained variance ranging from 11% to 40%. The weakest correlations were found in the training of physical fitness. This can be explained by the fact that physical fitness plays a subordinate role for performance in esports. Nevertheless, the majority of players (57%) are engaged in physical training. This result corresponds to the study of Froboese et al. [6], who found, that more than 50% of the esports players meet the physical activity recommendations of the World Health Organization.

The strongest correlations were found for the training of reaction time (explained variance: 26%) and repetitive movements (explained variance: 40%). These training areas are estimated as “somewhat important”. There is no clear reason for this result. It is surprising that high-estimated areas show a lower correlation between estimated and actual practice.

### RQ4 –the factor structure of esports competencies

The exploratory factor analysis revealed a six-factor solution of the 19 variables, that was interpreted as follows:

- Component 1: physical competencies (Conditioning)
- Component 2: sensori-motor or coordinative competencies
- Component 3: strategic-cognitive competencies
- Component 4: psychic competencies
- Component 5: social competencies
- Component 6: media-related competencies

The components are congruent with the integrative model of esports performance (reference) developed in the first part of the paper.

### RQ5 –structure of critical training components

For the estimated importance of 8 training areas, a three-factor solution was found. The factors were interpreted as follows:

- Component 1: speed of actions
- Component 2: strategy and psychophysical fitness
- Component 3: sensori-motor control

The components closely resemble the first three factors found in the estimated competence structure. The difference is that strategy and physical factors form a common component in contrast to the estimated competence structure. This could be explained by the fact that strategic actions can also depend on psychophysical endurance or fitness.

### RQ6 –structure of actual training

For the training engagement of 8 training areas, a two-factor solution was found. The factors were interpreted as follows:

- Component 1: speed and sensori-motor control
- Component 2: strategy and psychophysical fitness

For training engagement, the factor structure shows a lower dimensionality. The components of speed of actions and sensori-motor control found in RQ 5 are collapsed. Sensori-motor control and speed are closely related in esports, because fast movements often have to be executed very accurately and in the right order (e.g. mouse or finger movements). With regard to strategic action, a certain psychophysical endurance or fitness is always required in order to make the right decisions in the process of a game.

## Conclusion

This paper provides valuable insights into the training of esports players. In the first step, a performance model of esports based on game research and sports science was developed. In the second part, an online survey with 1,835 participants was performed. The survey revealed, that there are indeed differences between e-sports with regard to the importance and training of certain competencies and training areas. Among other things, insights were gained into the playing time, actual training time and break behaviour of esports players. In addition, factor structures of competencies, critical training components and actual training engagement were determined, which confirmed the performance model of esports developed in the first part of the paper. It was shown that many performance factors of sports can also be decisive in esports. Limitations of the work are due to the fact that it was not possible to ensure an even ratio of the players of the individual esports.

All in all, the work showed that esports deserve attention from the scientific community. Especially in the time of digitalization, a thorough understanding of esports training is important to develop and implement effective training as well as to discover possible deficits regarding health-related physical training. This study can be considered as a basis for future esports-specific training studies. Of course, future studies should address the specific structure of the different esports in more detail.

## Supporting information

S1 Data (SAV)

S1 File Survey. https://doi.org/10.6084/m9.figshare.12423842.
(DOCX)

S1 Table Means and standard deviations of H1 A. (DOCX)

S2 Table Means and standard deviations of H1 B. (DOCX)

S3 Table Means and standard deviations of H1 C. (DOCX)

S4 Table Mann-Whitney U-Tests H1 A. (DOCX)

S5 Table Mann-Whitney U-Tests H1 B. (DOCX)

S6 Table Means and standard deviations of H2. (DOCX)

S7 Table Mann-Whitney U-Tests H2 A. (DOCX)

S8 Table Mann-Whitney U-Tests H2 B. (DOCX)

S9 Table Means and standard deviations of H3. (DOCX)

S10 Table Mann-Whitney U-Tests H3 A. (DOCX)

S11 Table Mann-Whitney U-Tests H3 B. w
(DOCX)
