# Supplementary material for: The structure of performance and training in esports
Source: PLoS One. 2020 Aug 25;15(8):e0237584. doi: 10.1371/journal.pone.0237584 (PMC7447068; doi:10.1371/journal.pone.0237584)
Supplement: S1 File — https://doi.org/10.6084/m9.figshare.12423842. (DOCX) [file pone.0237584.s002.docx]

**S1 File. Survey.** <https://doi.org/10.6084/m9.figshare.12423842>
