# Supplementary material for: The structure of performance and training in esports
Source: PLoS One. 2020 Aug 25;15(8):e0237584. doi: 10.1371/journal.pone.0237584 (PMC7447068; doi:10.1371/journal.pone.0237584)
Supplement: S1 Table — (DOCX) [file pone.0237584.s003.docx]

S1 Table. Means and standard deviations of H1 A

| **A** | **Confidence** | | **Personal attitudes** | | **Dealing with pressure** | | **Motivation** | | **Analytical thinking** | | **Strategic thinking** | |
| --- | --- | --- | --- | --- | --- | --- | --- | --- | --- | --- | --- | --- |
|  | M | SD | M | SD | M | SD | M | SD | M | SD | M | SD |
| **SCII** | 4.06 | 0.794 | 4.24 | 0.949 | 4.74 | 0.623 | 4.33 | 0.862 | 4.31 | 0.778 | 4.32 | 0.727 |
| **RL** | 4.33 | 0.747 | 4.30 | 0.831 | 4.30 | 0.764 | 4.16 | 0.824 | 3.97 | 0.939 | 4.11 | 0.872 |
| **LoL** | 4.09 | 0.818 | 4.51 | 0.763 | 4.27 | 0.839 | 4.13 | 0.996 | 4.24 | 0.801 | 4.38 | 0.677 |
| **CS** | 4.48 | 0.693 | 4.49 | 0.708 | 4.51 | 0.659 | 4.31 | 0.787 | 4.20 | 0.806 | 4.31 | 0.777 |
| **FIFA** | 4.02 | 0.946 | 4.05 | 0.963 | 4.19 | 0.926 | 4.31 | 0.821 | 3.88 | 0.919 | 3.88 | 0.938 |
