# Supplementary material for: The structure of performance and training in esports
Source: PLoS One. 2020 Aug 25;15(8):e0237584. doi: 10.1371/journal.pone.0237584 (PMC7447068; doi:10.1371/journal.pone.0237584)
Supplement: S2 Table — (DOCX) [file pone.0237584.s004.docx]

S2 Table. Means and standard deviations of H1 B

| **B** | **Decision making** | | **Reaction time** | | **Accuracy** | | **Spatial orientation** | | **Eye-hand coordination** | | **Teamwork** | | **Acceptance of critical team feedback** | |
| --- | --- | --- | --- | --- | --- | --- | --- | --- | --- | --- | --- | --- | --- | --- |
|  | M | SD | M | SD | M | SD | M | SD | M | SD | M | SD | M | SD |
| **SCII** | 4.72 | 0.470 | 4.15 | 0.865 | 3.90 | 0.966 | 3.31 | 1.054 | 4.06 | 0.943 | 1.57 | 0.828 | 2.69 | 1.427 |
| **RL** | 4.82 | 0.439 | 4.26 | 0.874 | 4.30 | 0.753 | 4.34 | 0.813 | 4.14 | 0.955 | 4.52 | 0.720 | 4.07 | 0.970 |
| **LoL** | 4.65 | 0.647 | 4.12 | 0.884 | 3.89 | 0.862 | 3.67 | 0.997 | 4.00 | 0.914 | 4.44 | 0.801 | 4.04 | 1.132 |
| **CS** | 4.69 | 0.566 | 3.97 | 0.977 | 4.36 | 0.688 | 3.99 | 0.869 | 4.33 | 0.788 | 4.64 | 0.626 | 4.26 | 0.810 |
| **FIFA** | 4.02 | 0.868 | 4.09 | 0.996 | 3.84 | 0.988 | 3.52 | 0.822 | 3.98 | 1.017 | 2.38 | 1.182 | 3.00 | 1.108 |
