# Supplementary material for: The structure of performance and training in esports
Source: PLoS One. 2020 Aug 25;15(8):e0237584. doi: 10.1371/journal.pone.0237584 (PMC7447068; doi:10.1371/journal.pone.0237584)
Supplement: S3 Table — (DOCX) [file pone.0237584.s005.docx]

S3 Table. Means and standard deviations of H1 C

| **C** | **Ability to cope with**  **technical difficulties** | | **Adapting the game**  **Settings** | | **Physical strength** | | **Endurance** | | **Speed** | | **Agility** | |
| --- | --- | --- | --- | --- | --- | --- | --- | --- | --- | --- | --- | --- |
|  | M | SD | M | SD | M | SD | M | SD | M | SD | M | SD |
| **SCII** | 2.57 | 1.135 | 3.02 | 1.297 | 1.60 | 0.892 | 3.42 | 1.204 | 3.94 | 1.057 | 3.21 | 1.271 |
| **RL** | 3.29 | 1.134 | 3.30 | 1.146 | 1.57 | 0.828 | 2.72 | 1.238 | 3.16 | 1.453 | 2.69 | 1.364 |
| **LoL** | 3.10 | 1.209 | 3.24 | 1.272 | 1.77 | 1.039 | 2.94 | 1.297 | 2.73 | 1.319 | 2.48 | 1.255 |
| **CS** | 3.11 | 1.093 | 3.18 | 1.124 | 2.01 | 0.996 | 2.97 | 1.132 | 2.82 | 1.222 | 2.68 | 1.197 |
| **FIFA** | 3.48 | 1.064 | 3.88 | 1.044 | 2.84 | 1.335 | 3.40 | 1.242 | 3.55 | 1.366 | 3.34 | 1.384 |
