# Supplementary material for: The structure of performance and training in esports
Source: PLoS One. 2020 Aug 25;15(8):e0237584. doi: 10.1371/journal.pone.0237584 (PMC7447068; doi:10.1371/journal.pone.0237584)
Supplement: S4 Table — (DOCX) [file pone.0237584.s006.docx]

S4 Table. Mann-Whitney U-Tests H1 A

| Comparison 1-5 | Starcraft II  Rocket League | | Starcraft II  League of Legends | | Starcraft II  Counter Strike | | Starcraft II  FIFA | | Rocket League  League of Lgends | |
| --- | --- | --- | --- | --- | --- | --- | --- | --- | --- | --- |
|  | Z | p | Z | p | Z | p | Z | p | Z | p |
| Confidence | -3.724 | <0.001 | -0.431 | 0.666 | -6.144 | <0.001 | -0.080 | 0.936 | -3.222 | 0.001 |
| Personal attitudes | -0.235 | 0.814 | -2.339 | 0.019 | -2.581 | 0.010 | -1.585 | 0.113 | -2.854 | 0.004 |
| Dealing with pressure | -6.733 | <0.001 | -5.375 | <0.001 | -4.425 | <0.001 | -4.830 | <0.001 | -0.045 | 0.964 |
| Motivation | -2.609 | 0.009 | -1.656 | 0.098 | -0.677 | 0.499 | -0.318 | 0.750 | -0.454 | 0.650 |
| Analytical thinking | -3.615 | <0.001 | -0.628 | 0.530 | -1.437 | 0.151 | -3.000 | 0.003 | -2.900 | 0.004 |
| Strategic thinking | -2.253 | 0.024 | -0.561 | 0.575 | -0.114 | 0.910 | -3.045 | 0.002 | -3.015 | 0.003 |
| Decision making | -2.913 | 0.004 | -0.349 | 0.727 | -0.019 | 0.985 | -5.897 | 0.000 | -3.320 | 0.001 |
| Reaction time | -1.396 | 0.163 | -0.277 | 0.782 | -1.770 | 0.077 | -0.187 | 0.852 | -1.805 | 0.071 |
| Accuracy | -4.399 | <0.001 | -0.444 | 0.657 | -5.269 | <0.001 | -0.322 | 0.747 | -5.207 | <0.001 |
| Spatial orientation | -10.220 | <0.001 | -2.512 | 0.012 | -6.874 | <0.001 | -1.252 | 0.211 | -7.322 | <0.001 |
| Eye-hand coordination | -1.020 | 0.308 | -0.681 | 0.496 | -3.037 | 0.002 | -0.408 | 0.683 | -1.957 | 0.050 |
| Teamwork | -18.326 | <0.001 | -13.464 | <0.001 | -20.438 | <0.001 | -4.691 | <0.001 | -0.924 | 0.356 |
| Acceptance of critical feedback | -9.836 | <0.001 | -7.323 | <0.001 | -11.738 | <0.001 | -1.494 | 0.135 | -0.378 | 0.705 |
| Ability to cope w. technical issues | -6.192 | <0.001 | -3.495 | <0.001 | -5.120 | <0.001 | -4.978 | <0.001 | -1.715 | 0.086 |
| Adapting the game settings | -2.081 | 0.037 | -1.263 | 0.207 | -1.164 | 0.245 | -4.201 | <0.001 | -0.452 | 0.652 |
| Physical strength | -0.087 | 0.931 | -1.208 | 0.227 | -4.832 | <0.001 | -6.111 | <0.001 | -1.695 | 0.090 |
| Endurance | -5.523 | <0.001 | -2.919 | 0.004 | -4.025 | <0.001 | -0.003 | 0.998 | -1.730 | 0.084 |
| Speed | -5.312 | <0.001 | -7.070 | <0.001 | -9.280 | <0.001 | -1.543 | 0.123 | -3.160 | 0.002 |
| Agility | -3.919 | <0.001 | -4.403 | <0.001 | -4.462 | <0.001 | -0.840 | 0.401 | -1.463 | 0.143 |
