# Supplementary material for: The structure of performance and training in esports
Source: PLoS One. 2020 Aug 25;15(8):e0237584. doi: 10.1371/journal.pone.0237584 (PMC7447068; doi:10.1371/journal.pone.0237584)
Supplement: S5 Table — (DOCX) [file pone.0237584.s007.docx]

S5 Table. Mann-Whitney U-Tests H1 B

| Comparison 6-10 | Rocket League  Counter Strike | | Rocket League  FIFA | | League of Legends  Counter Strike | | League of Legends  FIFA | | Counter Strike  FIFA | |
| --- | --- | --- | --- | --- | --- | --- | --- | --- | --- | --- |
|  | Z | p | Z | p | Z | p | Z | p | Z | p |
| Confidence | -4.032 | <0.001 | -2.573 | 0.010 | -5.656 | <0.001 | -0.264 | 0.792 | -4.252 | <0.001 |
| Personal attitudes | -4.376 | <0.001 | -2.067 | 0.039 | -0.668 | 0.504 | -3.681 | <0.001 | -4.024 | <0.001 |
| Dealing with pressure | -5.310 | <0.001 | -0.619 | 0.536 | -2.932 | 0.003 | -0.478 | 0.632 | -2.606 | 0.009 |
| Motivation | -3.836 | <0.001 | -1.569 | 0.117 | -1.592 | 0.111 | -1.033 | 0.302 | -0.119 | 0.905 |
| Analytical thinking | -4.366 | <0.001 | -0.800 | 0.424 | -0.623 | 0.533 | -2.533 | 0.011 | -2.571 | 0.010 |
| Strategic thinking | -4.371 | <0.001 | -1.897 | 0.058 | -0.599 | 0.549 | -3.546 | <0.001 | -3.617 | <0.001 |
| Decision making | -4.915 | <0.001 | -9.932 | <0.001 | -0.440 | 0.660 | -5.429 | <0.001 | -7.373 | <0.001 |
| Reaction time | -5.718 | <0.001 | -1.143 | 0.253 | -1.446 | 0.148 | -0.045 | 0.964 | -0.979 | 0.327 |
| Accuracy | -1.117 | 0.264 | -3.602 | <0.001 | -6.180 | <0.001 | -0.037 | 0.970 | -4.184 | <0.001 |
| Spatial orientation | -8.342 | <0.001 | -7.182 | <0.001 | -3.521 | <0.001 | -0.945 | 0.345 | -4.132 | <0.001 |
| Eye-hand coordination | -3.378 | 0.001 | -1.230 | 0.219 | -4.170 | <0.001 | -0.139 | 0.889 | -2.696 | 0.007 |
| Teamwork | -3.484 | <0.001 | -11.983 | <0.001 | -2.924 | 0.003 | -9.345 | <0.001 | -13.602 | <0.001 |
| Acceptance of critical feedback | -3.306 | 0.001 | -6.941 | <0.001 | -1.291 | 0.197 | -5.727 | <0.001 | -8.563 | <0.001 |
| Ability to cope w. technical issues | -3.106 | 0.002 | -1.410 | 0.158 | -0.229 | 0.819 | -2.257 | 0.024 | -2.840 | 0.005 |
| Adapting the game settings | -2.048 | 0.041 | -3.849 | <0.001 | -0.534 | 0.593 | -3.274 | 0.001 | -4.762 | <0.001 |
| Physical strength | -9.380 | <0.001 | -7.557 | <0.001 | -3.241 | 0.001 | -5.221 | <0.001 | -4.824 | <0.001 |
| Endurance | -3.953 | <0.001 | -3.919 | <0.001 | -0.192 | 0.847 | -2.279 | 0.023 | -2.898 | 0.004 |
| Speed | -5.113 | <0.001 | -1.972 | 0.049 | -0.733 | 0.464 | -3.781 | <0.001 | -4.293 | <0.001 |
| Agility | -0.110 | 0.912 | -3.455 | 0.001 | -1.833 | 0.067 | -3.940 | <0.001 | -3.880 | <0.001 |
