# Supplementary material for: The structure of performance and training in esports
Source: PLoS One. 2020 Aug 25;15(8):e0237584. doi: 10.1371/journal.pone.0237584 (PMC7447068; doi:10.1371/journal.pone.0237584)
Supplement: S6 Table — (DOCX) [file pone.0237584.s008.docx]

S6 Table. Means and standard deviations of H2

| **TW** | **Reaction time** | | **Speed of single movements** | | **Repetitive moves** | | **Technique/**  **skills** | | **Movement**  **accuracy** | | **Strategy/**  **Tactics** | | **Stamina** | | **Physical fitness** | |
| --- | --- | --- | --- | --- | --- | --- | --- | --- | --- | --- | --- | --- | --- | --- | --- | --- |
|  | M | SD | M | SD | M | SD | M | SD | M | SD | M | SD | M | SD | M | SD |
| **SCII** | 3.64 | 1.092 | 3.68 | 1.056 | 3.75 | 1.200 | 4.39 | 0.843 | 4.08 | 0.802 | 4.51 | 0.656 | 3.72 | 0.976 | 2.54 | 1.007 |
| **RL** | 3.69 | 1.087 | 4.05 | 0.878 | 3.34 | 1.268 | 4.55 | 0.629 | 4.49 | 0.646 | 4.14 | 0.838 | 3.41 | 1.083 | 2.16 | 0.906 |
| **LoL** | 3.79 | 0.893 | 3.76 | 0.920 | 3.34 | 1.061 | 4.20 | 0.777 | 3.93 | 0.915 | 4.48 | 0.784 | 3.54 | 1.129 | 2.45 | 1.085 |
| **CS** | 3.64 | 1.072 | 3.74 | 0.872 | 3.04 | 1.136 | 4.18 | 0.806 | 4.23 | 0.771 | 4.39 | 0.700 | 3.63 | 1.007 | 2.72 | 1.001 |
| **FIFA** | 3.88 | 0.938 | 4.14 | 0.634 | 3.69 | 1.096 | 4.17 | 0.861 | 4.14 | 0.712 | 4.12 | 0.839 | 3.62 | 1.121 | 3.07 | 1.309 |
