# Supplementary material for: The structure of performance and training in esports
Source: PLoS One. 2020 Aug 25;15(8):e0237584. doi: 10.1371/journal.pone.0237584 (PMC7447068; doi:10.1371/journal.pone.0237584)
Supplement: S7 Table — (DOCX) [file pone.0237584.s009.docx]

S7 Table. Mann-Whitney U-Tests H2 A

| Comparison 1-5 | Starcraft II  Rocket League | | Starcraft II  League of Legends | | Starcraft II  Counter Strike | | Starcraft II  FIFA | | Rocket League  League of Legends | |
| --- | --- | --- | --- | --- | --- | --- | --- | --- | --- | --- |
|  | Z | p | Z | P | Z | p | Z | p | Z | p |
| Reaction time | -0.493 | 0.622 | -0.845 | 0.398 | -0.049 | 0.961 | -1.269 | 0.205 | -0.539 | 0.590 |
| Speed of single movements | -3.581 | <0.001 | -0.242 | 0.809 | -0.103 | 0.918 | -2.749 | 0.006 | -3.435 | 0.001 |
| Performing repetitive moves | -3.361 | 0.001 | -3.203 | 0.001 | -6.406 | <0.001 | -0.586 | 0.558 | -0.222 | 0.824 |
| Technique/skills | -1.536 | 0.125 | -2.436 | 0.015 | -3.281 | 0.001 | -1.878 | 0.060 | -4.979 | <0.001 |
| Movement accuracy | -5.568 | <0.001 | -1.196 | 0.232 | -2.077 | 0.038 | -0.325 | 0.745 | -6.761 | <0.001 |
| Strategy/tactics | -4.602 | <0.001 | -0.271 | 0.786 | -1.920 | 0.055 | -3.125 | 0.002 | -4.780 | <0.001 |
| Stamina | -2.845 | 0.004 | -1.002 | 0.316 | -0.842 | 0.400 | -0.350 | 0.726 | -1.461 | 0.144 |
| Physical fitness | -3.945 | <0.001 | -0.935 | 0.350 | -1.661 | 0.097 | -2.837 | 0.005 | -2.542 | 0.011 |
