# Supplementary material for: The structure of performance and training in esports
Source: PLoS One. 2020 Aug 25;15(8):e0237584. doi: 10.1371/journal.pone.0237584 (PMC7447068; doi:10.1371/journal.pone.0237584)
Supplement: S8 Table — (DOCX) [file pone.0237584.s010.docx]

S8 Table. Mann-Whitney U-Tests H2 B

| Comparison 6-10 | Rocket League  Counter Strike | | Rocket League  FIFA | | League of Legends  Counter Strike | | L. of Legends  FIFA | | Counter Strike  FIFA | |
| --- | --- | --- | --- | --- | --- | --- | --- | --- | --- | --- |
|  | Z | p | Z | P | Z | P | Z | P | Z | P |
| Reaction time | -0.999 | 0.318 | -1.085 | 0.278 | -1.184 | 0.236 | -0.672 | 0.502 | -1.531 | 0.126 |
| Speed of single movements | -7.043 | <0.001 | -0.319 | 0.749 | -0.180 | 0.857 | -2.753 | 0.006 | -3.514 | <0.001 |
| Performing repetitive moves | -4.866 | <0.001 | -1.943 | 0.052 | -2.888 | 0.004 | -2.101 | 0.036 | -4.190 | <0.001 |
| Technique/skills | -9.077 | <0.001 | -3.456 | 0.001 | -0.194 | 0.846 | -0.035 | 0.972 | -0.081 | 0.935 |
| Movement accuracy | -6.505 | <0.001 | -3.852 | <0.001 | -3.545 | <0.001 | -1.275 | 0.202 | -1.165 | 0.244 |
| Strategy/tactics | -5.663 | <0.001 | -0.125 | 0.901 | -2.255 | 0.024 | -3.251 | 0.001 | -2.407 | 0.016 |
| Stamina | -3.897 | <0.001 | -1.544 | 0.123 | -0.516 | 0.606 | -0.424 | 0.672 | -0.158 | 0.875 |
| Physical fitness | -10.579 | <0.001 | -5.386 | <0.001 | -2.956 | 0.003 | -3.172 | 0.002 | -2.395 | 0.017 |
