# Supplementary material for: The structure of performance and training in esports
Source: PLoS One. 2020 Aug 25;15(8):e0237584. doi: 10.1371/journal.pone.0237584 (PMC7447068; doi:10.1371/journal.pone.0237584)
Supplement: S9 Table — (DOCX) [file pone.0237584.s011.docx]

S9 Table. Means and standard deviations of H3

|  | **Reaction time** | | **Speed of single movements** | | **Repetitive moves** | | **Technique/**  **skills** | | **Movement**  **accuracy** | | **Strategy/**  **Tactics** | | **Stamina** | | **Physical fitness** | |
| --- | --- | --- | --- | --- | --- | --- | --- | --- | --- | --- | --- | --- | --- | --- | --- | --- |
|  | M | SD | M | SD | M | SD | M | SD | M | SD | M | SD | M | SD | M | SD |
| **SCII** | 2.72 | 1.180 | 3.17 | 1.187 | 3.31 | 1.226 | 3.74 | 1.019 | 3.35 | 1.163 | 4.05 | 1.111 | 2.80 | 1.162 | 2.90 | 1.299 |
| **RL** | 2.69 | 1.197 | 3.63 | 1.080 | 3.07 | 1.377 | 4.35 | 0.828 | 4.03 | 0.981 | 3.30 | 1.159 | 2.40 | 1.123 | 2.62 | 1.270 |
| **LoL** | 2.70 | 1.166 | 2.94 | 1.247 | 2.88 | 1.344 | 3.46 | 1.216 | 3.16 | 1.285 | 3.75 | 1.267 | 2.88 | 1.338 | 2.87 | 1.388 |
| **CS** | 2.70 | 1.211 | 3.17 | 1.169 | 2.78 | 1.265 | 3.67 | 1.101 | 3.61 | 1.139 | 3.39 | 1.202 | 2.63 | 1.176 | 2.90 | 1.229 |
| **FIFA** | 2.53 | 1.287 | 2.88 | 1.171 | 2.81 | 1.177 | 3.26 | 1.163 | 3.03 | 1.311 | 3.34 | 1.278 | 2.55 | 1.111 | 2.62 | 1.240 |
