# Supplementary material for: The structure of performance and training in esports
Source: PLoS One. 2020 Aug 25;15(8):e0237584. doi: 10.1371/journal.pone.0237584 (PMC7447068; doi:10.1371/journal.pone.0237584)
Supplement: S10 Table — (DOCX) [file pone.0237584.s012.docx]

S10 Table. Mann-Whitney U-Tests H3 A

| Comparison 1-5 | Starcraft II  Rocket League | | Starcraft II  League of Legends | | Starcraft II  Counter Strike | | Starcraft II  FIFA | | Rocket League  League of Legends | |
| --- | --- | --- | --- | --- | --- | --- | --- | --- | --- | --- |
|  | Z | p | Z | p | Z | p | Z | p | Z | p |
| Reaction time | -0.219 | 0.826 | -0.068 | 0.946 | -0.270 | 0.787 | -1.030 | 0.303 | -0.138 | 0.890 |
| Speed of single movements | -3.984 | <0.001 | -1.543 | 0.123 | -0.033 | 0.974 | -1.518 | 0.129 | -5.873 | <0.001 |
| Performing repetitive moves | -1.704 | 0.088 | -2.617 | 0.009 | -4.338 | <0.001 | -2.623 | 0.009 | -1.436 | 0.151 |
| Technique/skills | -6.637 | <0.001 | -1.600 | 0.110 | -0.439 | 0.660 | -2.718 | 0.007 | -8.310 | <0.001 |
| Movement accuracy | -6.172 | <0.001 | -1.100 | 0.271 | -2.432 | 0.015 | -1.533 | 0.125 | -7.258 | <0.001 |
| Strategy/tactics | -6.624 | <0.001 | -1.877 | 0.061 | -5.858 | <0.001 | -3.601 | <0.001 | -4.292 | <0.001 |
| Stamina | -3.632 | <0.001 | -0.533 | 0.594 | -1.478 | 0.139 | -1.325 | 0.185 | -3.862 | <0.001 |
| Physical fitness | -2.075 | 0.038 | -0.209 | 0.835 | -0.198 | 0.843 | -1.181 | 0.238 | -1.802 | 0.072 |
