# Supplementary material for: The structure of performance and training in esports
Source: PLoS One. 2020 Aug 25;15(8):e0237584. doi: 10.1371/journal.pone.0237584 (PMC7447068; doi:10.1371/journal.pone.0237584)
Supplement: S11 Table — w (DOCX) [file pone.0237584.s013.docx]

S11 Table. Mann-Whitney U-Tests H3 B

| Comparison 6-10 | Rocket League  Counter Strike | | Rocket League  FIFA | | League of Legends  Counter Strike | | League of Legends  FIFA | | Counter Strike  FIFA | |
| --- | --- | --- | --- | --- | --- | --- | --- | --- | --- | --- |
|  | Z | p | Z | P | Z | P | Z | P | Z | P |
| Reaction time | -0.109 | 0.913 | -1.028 | 0.304 | -0.224 | 0.823 | -0.983 | 0.326 | -1.016 | 0.310 |
| Speed of single movements | -7.550 | <0.001 | -4.673 | <0.001 | -2.051 | 0.040 | -0.208 | 0.836 | -1.762 | 0.078 |
| Performing repetitive moves | -4.147 | <0.001 | -1.517 | 0.129 | -0.737 | 0.461 | -0.258 | 0.797 | -0.180 | 0.857 |
| Technique/skills | -12.379 | <0.001 | -7.342 | <0.001 | -1.689 | 0.091 | -1.295 | 0.195 | -2.758 | 0.006 |
| Movement accuracy | -7.145 | <0.001 | -5.801 | <0.001 | -3.773 | <0.001 | -0.638 | 0.523 | -3.361 | 0.001 |
| Strategy/tactics | -1.740 | 0.082 | -0.341 | 0.733 | -3.441 | 0.001 | -2.168 | 0.030 | -0.276 | 0.782 |
| Stamina | -3.966 | <0.001 | -1.136 | 0.256 | -2.018 | 0.044 | -1.596 | 0.110 | -0.460 | 0.646 |
| Physical fitness | -4.314 | <0.001 | -0.085 | 0.932 | -0.367 | 0.713 | -1.064 | 0.287 | -1.595 | 0.111 |
